# Loss of *Fis1* impairs proteostasis during skeletal muscle aging in *Drosophila*

**DOI:** 10.1111/acel.13379

**Published:** 2021-06-01

**Authors:** Tai‐Ting Lee, Po‐Lin Chen, Matthew P. Su, Jian‐Chiuan Li, Yi‐Wen Chang, Rei‐Wen Liu, Hsueh‐Fen Juan, Jinn‐Moon Yang, Shih‐Peng Chan, Yu‐Chen Tsai, Sophia von Stockum, Elena Ziviani, Azusa Kamikouchi, Horng‐Dar Wang, Chun‐Hong Chen

**Affiliations:** ^1^ National Institute of Infectious Diseases and Vaccinology National Health Research Institutes Zhunan Taiwan; ^2^ Division of Biological Science Graduate School of Science Nagoya University Nagoya Japan; ^3^ Institute of Biotechnology National Tsing Hua University Hsinchu Taiwan; ^4^ National Mosquito‐Borne Diseases Control Research Center National Health Research Institutes Zhunan Taiwan; ^5^ Department of Life Science National Taiwan University Taipei Taiwan; ^6^ Department of Life Science and Life Science Center Tunghai University Taichung Taiwan; ^7^ Institute of Bioinformatics and Systems Biology National Chiao Tung University Hsinchu Taiwan; ^8^ Graduate Institute of Microbiology College of Medicine National Taiwan University Taipei Taiwan; ^9^ Genome and Systems Biology Degree Program College of Life Science National Taiwan University Taipei Taiwan; ^10^ Department of Biology University of Padova Padova Italy; ^11^ Fondazione Ospedale San Camillo IRCCS, Lido di Venezia Venezia Italy

**Keywords:** aging, *Drosophila melanogaster*, Fis1, mitochondria

## Abstract

Increased levels of dysfunctional mitochondria within skeletal muscle are correlated with numerous age‐related physiopathological conditions. Improving our understanding of the links between mitochondrial function and muscle proteostasis, and the role played by individual genes and regulatory networks, is essential to develop treatments for these conditions. One potential player is the mitochondrial outer membrane protein Fis1, a crucial fission factor heavily involved in mitochondrial dynamics in yeast but with an unknown role in higher‐order organisms. By using *Drosophila melanogaster* as a model, we explored the effect of Fis1 mutations generated by transposon Minos‐mediated integration. Mutants exhibited a higher ratio of damaged mitochondria with age as well as elevated reactive oxygen species levels compared with controls. This caused an increase in oxidative stress, resulting in large accumulations of ubiquitinated proteins, accelerated muscle function decline, and mitochondrial myopathies in young mutant flies. Ectopic expression of Fis1 isoforms was sufficient to suppress this phenotype. Loss of *Fis1* led to unbalanced mitochondrial proteostasis within fly muscle, decreasing both flight capabilities and lifespan. Fis1 thus clearly plays a role in fly mitochondrial dynamics. Further investigations into the detailed function of Fis1 are necessary for exploring how mitochondrial function correlates with muscle health during aging.

## Graphical abstract

Ubiquitinated proteins and autophagic proteins accumulate within *Drosophila melanogaster* muscle fibers as they age. Here we generated *Drosophila* mutants for the gene Fis1, which influences mitochondrial dynamics, and observed similar phenotypes in these transgenic lines. The unbalanced proteostasis resulting from Fis1 mutation led to muscle fiber degeneration and reduced health spans. Fis1‐related mitochondrial quality control is an important factor in determining Drosophila muscle, and thus overall, health.

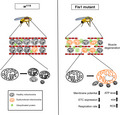

## 1 INTRODUCTION

The progressive decline in skeletal muscle strength and mass is a major cause of frailty during aging (Vainshtein et al., 2014). Studies have linked these declines to the dysregulation of proteostasis during aging (Labbadia & Morimoto, 2015). Although the process through which this change occurs remains debatable, redox‐related regulation pathways are considered the likely cause of age‐related changes in metabolism (Fernando et al., 2019). Mitochondria are the main source of cellular reactive oxygen species (ROS), which are by‐products of the electron transport chain (ETC; Ábrigo et al., 2018; Zorov et al., 2014). Compared with other tissues, skeletal muscle requires more adenosine triphosphate (ATP) for energy production and contains a larger number of mitochondria; mitochondria therefore heavily influence levels of oxidative stress in skeletal muscle (Ábrigo et al., 2018). Mitochondrial dysfunction can lead to increased ROS production, a hallmark of neurodegenerative and age‐related diseases such as Alzheimer's disease (AD; Wong‐Riley et al., 1997) and Parkinson's disease (Henchcliffe & Beal, 2008). Identifying the mechanisms that disrupt proteostasis and/or mitochondrial function in aging animals might thus lead to treatments that prevent disease incidence and delay age‐related illness.

Mitochondrial dynamics (fission/fusion) and mitophagy are two closely related mechanisms (Ashrafi & Schwarz, 2013; Twig & Shirihai, 2011) responsible for maintaining a healthy mitochondrial pool and eliminating damaged mitochondria, respectively (Lo Verso et al., 2014; Twig et al., 2008; Vainshtein et al., 2014). Mitochondrial fission and fusion processes are regulated by large guanosine triphosphatases (GTPases) of the dynamin family (Chan, 2012; Kraus & Ryan, 2017; Ni et al., 2015; Pernas & Scorrano, 2016; Tilokani et al., 2018; Westermann, 2010), which are highly conserved in yeast, flies, and mammals (Hales & Fuller, 1997; Hoppins et al., 2007; Liesa et al., 2009; Zhao et al., 2013). Mitochondrial fusion in mammals is mediated by the fusion proteins mitofusin (Mfn) 1, Mfn2, and optic atrophy 1 (OPA1); on the other hand, fission processes are mainly mediated by dynamin‐related protein 1 (Drp1; Kraus & Ryan, 2017; Ni et al., 2015; Pernas & Scorrano, 2016).

The first step of mitophagy‐mediated quality control is the segregation of healthy and damaged mitochondria, with damaged mitochondria possessing depolarized mitochondrial membrane potential (Ashrafi & Schwarz, 2013; Jin & Youle, 2012; Twig & Shirihai, 2011). Studies have reported that Mfn1 and Mfn2 are ubiquitinated during PINK1/parkin‐dependent mitophagy, resulting in the removal of damaged mitochondria in mammals and flies (Gegg et al., 2010; Geisler et al., 2010; Poole et al., 2010). As previously mentioned, Drp1, the mammalian homolog of the yeast Dnm1, is essential for mitophagy (Mao & Klionsky, 2013; Twig et al., 2008). As such, the core proteins that regulate mitochondrial dynamics also influence mitochondrial proteostasis; however, the interplay between mitochondrial dynamics and proteostasis during aging in muscle remains poorly understood.

*Fis1*, a crucial fission factor involved in the recruitment of Dnm1, is located on the mitochondrial outer membrane in yeast and may play a role in modulating mitochondrial dynamics (Mozdy et al., 2000; Shen et al., 2014). Although *Fis1* is evolutionarily conserved from yeasts to humans, its direct participation in metazoan fission machinery remains debated (Hoppins et al., 2007; Okamoto & Shaw, 2005; Otera et al., 2010; Suzuki et al., 2003; Yu et al., 2019). Moreover, *Fis1* knockout studies reported decreased mitophagy (Twig et al., 2008) and have suggested that *Fis1* controls mitophagy through a regulated ER‐resident SNARE (soluble N‐ethylmaleimide‐sensitive factor attachment protein receptor) protein (Xian et al., 2019). Consistent with this, *Fis1* has been reported to fragment mitochondria and enhance the formation of mitochondria‐targeting autophagosomes (Gomes & Scorrano, 2008). *Fis1* thus appears related to mitochondrial dynamics and mitophagy, although the mechanisms through which *Fis1* regulates tissue‐specific mitochondrial dynamics and proteostasis in an age‐dependent manner are unknown.

Here, we used *Drosophila melanogaster* (*D*. *melanogaster*) as a model for studying *Fis1*‐mediated mitochondrial function and proteostasis in muscles. The transcription of *Fis1* results in the production of at least six mRNA isoforms in *D*. *melanogaster*, denoted as C (698 bp), E (628 bp), A (630 bp), G (557 bp), D (560 bp), and F (704 bp) forms. After post‐translation modification, the isoforms translate into four Fis1 polypeptides: C (154 amino acids); E (148 amino acids); A and G (both with 98 amino acids); and D and F (both with 92 amino acids).

To simplify the nomenclature, we divided the isoforms into two groups, one for longer forms (more than 100 amino acids) and one for shorter forms (<100 amino acids). Next, we further subdivided each group according to isoform length. Both C and E forms were thus designated as long forms because they each contain more than 100 aa. However, because the C form (154 aa) is longer than the E form (148 aa), we denoted the C form as long form long (LL) and the E form as long form short (LS). Similarly, we designated the A and G forms as short form long (SL) and the D and F forms as short form short (SS).

By generating Fis1‐specific antibodies, we observed that the expression of the two longer forms increased with age. Loss of *Fis1* led to mitochondrial enlargement and degeneration of mitochondrial inner membranes in both young and old flies. These *Fis1*‐lacking mitochondria exhibited multiple signatures of dysfunction, including decreased ETC complex protein levels, increased mitochondrial ROS leakage, and depolarized membrane potential. Moreover, by using *Fis1* mutant flies, we found that the depletion of *Fis1* dysregulated proteostasis and caused ubiquitinated protein accumulation within muscle fibers. *Fis1* mutant flies also exhibited significantly reduced lifespans and mobility compared with controls. Our findings reveal that elevated ROS levels result in functional muscle decline, thus reducing proteostasis and ultimately leading to flight‐muscle degeneration. These results suggest that loss of *Fis1* can lead to a decline in mitochondria function and accelerate muscle function decline.

## 2 MATERIALS AND METHODS

### 2.1 *Drosophila* rearing

Fly stocks were reared at 25 ± 1°C using commercial food (WellGenetics) on a 12‐h light/12‐h dark (LD 12:12) entrainment regime. All fly stocks were obtained from the Bloomington *Drosophila* Stock Center, *Drosophila* Genomics Resource Center, or were generated within our lab. Stock details are in key resource table (Table 1, Table 2).

**TABLE 1:** Detailed information of reagents

Reagent or resource	Source	Identifier
Antibodies		
Anti‐rabbit porin	Abcam, Cambridge, UK	#14734
Anti‐mouse GAPDH	GeneTex, Hsinchu, Taiwan	#627408
Anti‐mouse tubulin	GeneTex, Hsinchu, Taiwan	#628802
Anti‐rabbit GFP	Abcam, Cambridge, UK	#6556
Anti‐ mouse HA	Biolegend, USA	#901514
Anti‐mouse HA	Merck Millipore	#05‐904
Anti‐rabbit *Fis1*	This paper	N/A
Anti‐rabbit ubiquitin	Cell Signaling Technology, Danvers, MA, USA	#3933S
Anti‐Atg8a	Millipore	ABC974‐1
Anti‐Ref(2)p	Abcam	ab178440
Anti‐NDUFS3	Abcam	ab110246
Anti‐chchd3	A gift from Dr. Chao Tong	N/A
HRP‐conjugated goat anti‐rabbit IgG polyclonal antibody	GeneTex, Hsinchu, Taiwan	GTX213110‐02
HRP‐conjugated goat anti‐mouse IgG polyclonal antibody	GeneTex, Hsinchu, Taiwan	GTX213110‐01
Anti‐FK2	Merck Millipore	#04‐263
Anti‐rabbit 4‐HNE	Abcam, Cambridge, UK	#46545
Anti‐mouse ATP5a	Abcam, Cambridge, UK	#14748
Alex Flour 488 anti‐mouse	Thermo Fisher Scientific	Cat# A‐11029
Alex Flour 647 anti‐rabbit	Thermo Fisher Scientific	Cat # A32733
Chemicals, peptides, and recombinant proteins		
VectaShield Antifade Mounting Medium	Vector Laboratories, Burlingame, CA, USA	# HV‐93952‐25
Phalloidin	Sigma‐Aldrich, St. Louis, MO, USA	P1951
Critical commercial assays		
MitoSOX^™^	Thermo Fisher Scientific	Cat# M36008
MitoTracker^®^ Green	Thermo Fisher Scientific	Cat# M7514
JC‐1 Dye	Thermo Fisher Scientific	#T3168
XCell™ SureLock™ Mini‐Cell	Thermo Fisher Scientific	#EI0001
NativePAGE™ Running Buffer Kit	Thermo Fisher Scientific	#BN2007
NativePAGE™ Sample Prep Kit	Thermo Fisher Scientific	#BN2008
Colloidal Blue Staining Kit	Thermo Fisher Scientific	#LC6025
NativeMark™ Unstained Protein Standard	Thermo Fisher Scientific	#LC0725
ATP Bioluminescence Assay kit CLS II	Roche	#Cat 1699695
Digitonin	Sigma‐Aldrich, St. Louis, MO, USA	#D141
Experimental models: organisms/strains		
Mef2‐GAL4	Bloomington *Drosophila* Stock Center	#50742
*white* ^1118^	Bloomington *Drosophila* Stock Center	#3605
*Fis1* ^MI10520^	Bloomington *Drosophila* Stock Center	#55496
*gstD*‐GFP	(Sykiotis et al., 2008)	N/A
*gstD*‐GFP‐*Fis1*mutant	N/A	N/A
UAS‐miR‐LL	N/A	N/A
UAS‐miR‐LL	N/A	N/A
UAS‐attB‐LL‐3xHA	N/A	N/A
UAS‐attB‐LS‐3xHA	N/A	N/A
Oligonucleotides		
Rp49_Q‐PCR_F	N/A	TAAGCTGTCGCACAAATGGC
Rp49_Q‐PCR_R	N/A	GCACCAGGAACTTCTTGAAT
*Fis1*‐C/E_Q‐PCR‐F	N/A	ATCACGAACTTGAGTTGGATGGTG
*Fis1*‐C/E_Q‐PCR‐R	N/A	GTACCGACTTCGGACCAGAC
*Fis1*‐A/D_Q‐PCR‐F	N/A	TTGTATTTGCTAACAACATTTGCC
*Fis1*‐A/D_Q‐PCR‐R	N/A	AGAATGCGTACTTCTAAATCTTC
*Fis1*‐F/G_Q‐PCR‐F	N/A	TTCTTCTCAATTCTAATGACACAAT
*Fis1*‐F/G_Q‐PCR‐R	N/A	ATAGTCACGCCTTCCATCTG
nuc‐tub Q‐PCR F1	N/A	TATAAGTAAAGGCAGCAGGGAGAC
nuc‐tub Q‐PCR R1	N/A	ATCTGGGTACTCTTCCTCTCCATC
mt‐ND5 Q‐PCR F1	N/A	GAAGTAAAGCTACATCCCCAATTCG
mt‐ND5 Q‐PCR R1	N/A	GGTGAGATGGTTTAGGACTTGTTTC
mt‐CytB Q‐PCR‐F1	N/A	TAGTGTTAATCATATTTGTCGAGACGTT
mt‐CytB Q‐PCR‐R1	N/A	ATATGAACCGTAATAAATTCCTCGTCC
mt‐CoI Q‐PCR‐F1	N/A	ATTAGGTGCTCCTGATATAGC
mt‐CoI Q‐PCR‐R1	N/A	CAATTCCAGCGGATAGAGGTGG
Recombinant DNA		
*Fis1*‐miR‐LL‐1	This paper	GGCAGCTTACTTAAACTTAATCACAGCCTTTAATGTAACGGGAGAAATAGTAACGTCATAAGTTAATATACCATATC
*Fis1*‐miR‐LS‐2	This paper	AATAATGATGTTAGGCACTTTAGGTACAACACGTTTTTAACCGACAATGTAGATATGGTATATTAACTTACATT
Mir6.1_5’EcoRI/BglII	This paper	GGCGAATTCCGCCAGATCTTTTAAAGTCCACAACTCATCAAGGAAAATGAAAGTCAAAGTTGGCAGCTTACTTAAACTTA
Mir6.1_3′NotI/BamHI	This paper	GGCCGCGGCCGCACGGATCCAAAACGGCATGGTTATTCGTGTGCCAAAAAAAAAAAAAATTAAATAATGATGTTAGGCAC
pUAST‐attB‐3xHA fusion *Fis1*‐CDS_F	This paper	AGGGAATTGGGAATTCATGGAGGATCTTTTAAACGAAGTTGTACCA
pUAST‐attB‐3xHA fusion *Fis1*‐LL‐CDS_R	This paper	CGTATGGGTACTCGAGTTTCTCCCGTTTTTGTTTATTTCTAGCCAT
pUAST‐attB‐3xHA fusion *Fis1*‐LS‐CDS_R	This paper	CGTATGGGTACTCGAGCTTTCTAGCCATAGCAATGCCAAGTCCTAA

**TABLE 2:** Abbreviation list

Abbreviation	Name
4HNE	4‐Hydroxynonenal
AD	Alzheimer's disease
AGC	Automated gain control
Atg8a	Autophagy‐related protein 8a
ATP5a	ATP synthase alpha‐subunit
BN‐PAGE	Blue Native‐polyacrylamide gel electrophoresis
Chchd3	Coiled‐Coil‐Helix‐Coiled‐Coil‐Helix Domain Containing 3
Dnm1	Dynamin 1
Drp1	Dynamin‐related protein 1
DSM	Drosophila Schneider's Medium
ETC	Electron transport chain
FCCP	Carbonyl cyanide 4‐(trifluoromethoxy)phenylhydrazone
Fis1	Fission, mitochondrial 1
Fis1‐LL	Fission, mitochondrial 1‐Long form long
Fis1‐LS	Fission, mitochondrial 1‐Long form short
Fis1‐SL	Fission, mitochondrial 1‐Short form long
Fis1‐SS	Fission, mitochondrial 1‐Short form short
GAPDH	Glyceraldehyde‐3‐phosphate dehydrogenase
gstD	Glutathione S transferase D1
KEGG	Kyoto Encyclopedia of Genes and Genomes
LC‐MS/MS	Liquid chromatography‐tandem mass spectrometry
Marf	Mitochondrial associated regulatory factor
Mef2	Myocyte enhancer factor‐2
Mfn	Mitofusin
NDUFS3	NADH:Ubiquinone Oxidoreductase Core Subunit S3
OPA1	Optic atrophy type 1
OsO4	Osmium tetroxide
PBS	Phosphate‐Buffered Saline
PBST	1X Phosphate‐Buffered Saline containing 0.1% Triton X‐100
PINK1	PTEN‐induced kinase 1
RCR	Respiratory control ratios
ROS	Reactive oxygen species
TPR	Tetratricopeptide repeat

### 2.2 Lifespan assay

Control *w* ^1118^ and *Fis1* mutant flies were collected within 24 h of eclosion and then maintained at 25°C in vials containing commercial fly food under an LD 12:12 cycle. Flies were provided fresh food every 3–4 days. Each vial contained 25 male or non‐virgin female flies, and different sexes and genotypes were housed separately. The sex ratio was maintained at 1:1 for experiments. Flies were counted at each transfer until all had died.

### 2.3 Behavioral assays

#### 2.3.1 Climbing

Flies were maintained at 25°C in vials containing standard fly food. Each experimental repetition used 10–20 wild‐type or mutant flies. Flies were anesthetized using CO_2_ before being placed in vials at least 24 h prior to the start of each assay to reduce potential side effects. Each tube contained a maximum of 25 flies of the same sex.

Flies were then loaded into the first tube of a countercurrent apparatus without CO_2_. The flies were tapped down to the bottom of the tube and then allowed to climb into the top receiver tube for 30 s. Flies that climbed into the top tube were then forced into a second tube. This was repeated five times per assay. The distribution of flies between the tubes was counted each time to calculate the distribution ratio and performance index (PI). The tubes were scored from 0 to 5, left to right.

The distribution ratio was defined as:

$$\frac{\text{Number of flies in tube}}{\text{Total number of flies}}\times 100$$

The PI was defined as:

$$\frac{\sum_{i=0}^{5}i\times \text{number of flies per tube}}{\text{Total number of flies}\times 5}\times 100$$

where *i* is the score of each tube.

#### 2.3.2 Flight duration

Flies were fully anesthetized on ice and then gently placed dorsal side up on a cold Petri dish. The tip of a tether (copper wire) was gently dipped into wood glue before being placed onto the fly's head. The angle between the tether and the fly was approximately 30°, and care was taken to avoid disturbing the wing vibration. Fixed flies were then placed in a constant‐temperature environment (28°C) for 1 h in groups of 10 to allow for full recovery from the ice anesthesia. Next, fixed flies were set in the same orientation and puffed gently with air before immediately being filmed with a camera for 120 s. The total recorded flying time for each fly was estimated by counting the total time each individual spent during video playback. A minimum of 30 flies were tested per genotype in batches of 5–10.

### 2.4 Western blot

Protein was extracted from the thoraxes of 5–7‐day‐old male flies and was then homogenized in 1× RIPA buffer (20 mM Tris–HCl; pH 7.5, 150 mM NaCl; 1 mM Na_2_ EDTA; 1 mM EGTA; 1% NP‐40; 1% sodium deoxycholate; 2.5 mM sodium pyrophosphate; 1 mM Na_3_VO_4_; 1 mM β‐glycerophosphate), 10% protease inhibitor, and 4% phosphatase inhibitor. Samples were then centrifuged at 16,100 *g* for 10 min at 4°C and separated by sodium dodecyl sulfate–polyacrylamide gel electrophoresis (PAGE). Next, proteins were transferred to a PVDF membrane, which was probed with the following antibodies: anti‐GFP (1:10,000; Abcam), anti‐HA (1:10,000; Biolegend), anti‐*Fis1* (1:10,000), anti‐GAPDH (1:10,000; GeneTex), anti‐tubulin (1:10,000; GeneTex), porin (1:10,000; Abcam), anti‐ubiquitin (1:10,000; Cell Signaling Technology), anti‐ATP5a (1:10,000; Abcam), anti‐NDUFS3 (1:10,000; Abcam), and anti‐Chchd3 (1:10,000; gifted by Dr. Chao Tong). Membranes were then incubated with mouse or rabbit peroxidase‐conjugated secondary antibody. Blots were detected using ECL Western Blotting Substrate (Merck Millipore), and images were analyzed using the ImageJ software package.

### 2.5 Adult muscle dissection and immunofluorescence

The thoracic muscle of male flies was dissected in phosphate‐buffered saline (PBS 1.37 M NaCl, 27 mM KCl, 4 mM Na_2_HPO_4_, 17.6 mM KH_2_PO_4_; pH 7.4). First, an incision was made between the fly's legs, and then, half of the thorax was washed in PBS buffer. The thoracic muscles were then fixed for 30 min in 4% paraformaldehyde (Sigma‐Aldrich)–1× PBS at room temperature (25°C). After fixation, the supernatant was removed and 0.1% PBST (0.1% triton in 1× PBS) was used to wash the muscle tissue three times for 10 min each time. Muscle tissue was blocked with 2% bovine serum albumin (BSA)/0.1% PBST for 2 h at room temperature. The solution was then removed, and the tissues were washed three times in 0.1% PBST for 10 min. The tissue was then incubated with the respective primary antibody, which was diluted using 2% BSA/0.1% PBST overnight at 4℃. Muscle was probed with the following antibodies: anti‐FK2 (Merck Millipore), anti‐HA (1:400; Abcam), and anti‐ATP5a (1:400; Abcam). The next day, the muscle tissue was washed three times for 10 min in 0.1% PBST. The secondary antibody and phalloidin (Sigma‐Aldrich) were then added to the muscle tissues, which were kept at room temperature (25°C) for 2–4 h. The Alexa Fluor 488 anti‐mouse (Thermo Fisher Scientific) and Alexa Fluor 647 anti‐rabbit (Thermo Fisher Scientific) secondary antibodies were used. Subsequent to this, the samples were washed three times in 0.1% PBST and mounted in VectaShield antifade mounting medium (Vector Laboratories). Images were captured using a laser confocal microscope (Leica SP5) with a hybrid detector (HyD). Images were analyzed and quantified using ImageJ.

### 2.6 JC‐1 staining

Adult flight muscles were dissected in Schneider's medium (DSM) and then incubated in 2 µM JC‐1 solution (diluted in DSM) for 45 min at room temperature. The muscles were then immediately mounted in DSM and imaged using the Leica SP5 laser confocal microscope. The intensity of fluorescent aggregates was quantified using ImageJ.

### 2.7 MitoSox staining

Adult flight muscles were dissected in DSM. Hemithoraxes were incubated in staining solution (5 μM MitoSox™ Red [M36008, Thermo Fisher Scientific] and 100 nM MitoTracker Green [M7514, Thermo Fisher Scientific]) for 12 min at room temperature. The hemithoraxes were washed twice with DSM for 30 s at room temperature. Samples were mounted in DSM solution. Slides were then imaged on the Leica SP5 laser confocal microscope using identical settings for each condition. MitoSox intensity was quantified using Image J.

### 2.8 Mitochondrial respiration analysis

Mitochondrial respiration rates were measured using an Oxytherm System (Hansatech) with magnetic stirring, and thermostatic control was maintained at 25°C. Isolated mitochondria (1 mg/ml) from *D*. *melanogaster* were incubated in 120 mM KCl, 5 mM Pi‐Tris, 3 mM HEPES, 1 mM EGTA, and 1 mM MgCl_2_ (pH 7.2). Oxygen consumption was calculated according to the slope of the registered graph and plotted as Ng atoms: O_2_ × min^−1^ × mg^−1^. Respiratory control ratios (RCRs; adenosine diphosphate [ADP]‐stimulated respiration over basal respiration) and maximal respiration (FCCP‐stimulated respiration over basal respiration) were calculated (Von Stockum et al., 2019).

### 2.9 Plasmid construction

#### 2.9.1 pUAST‐attB_miR‐Fis1‐LL or miR‐LS vector

After alignment analysis of all of the *Fis1* alternative splicing mRNA sequences, each highly specific sequence with 22 contiguous nucleotides was identified for miR‐based RNAi design and located within the 3′ UTR region between the *Fis1*‐LL (*Fis1*‐C type) and *Fis1*‐LS (*Fis1*‐E type) isoform RNA transcripts (S1B). The functional stem–loop structure of the artificial mir‐based *RNAi_Fis1*‐*LL* and *Fis1*‐*LS* miRNAs was generated through the first primer set_ *Fis1*‐*miR*‐*LL* or *Fis1*‐*miR*‐*LS* primers in a polymerase chain reaction (PCR). The functional miRNAs were extended and flanking sequences added with restriction enzyme sites by the second primer set_Mir6.1_5′EcoRI/BglII and Mir6.1_3′NotI/BamHI primers to obtain precursor *Fis1*‐*miRNA* units (Chen et al., 2007). The restriction enzyme double‐digested EcoRI/NotI‐*Fis1*‐*miRs* were integrated into the EcoRI and NotI site region of the pUAST‐attB vector (GenBank: EF362409.1) to generate the pUAST‐attB_*miR*‐*Fis1*‐*LL* and pUAST‐attB_*miR*‐*Fis1*‐*LS* plasmids.

#### 2.9.2 pUAST‐attB_Fis1‐3xHA vector

*Fis1*‐CDS PCR fragments without stop codons were generated through a PCR of the cDNA pools from the *D*.* melanogaster w* ^1118^ strain by using a pUAST‐attB_3xHA fusion *Fis1*‐CDS primer set. Agarose gel‐purified PCR products of *Fis1*‐CDS fragments were cloned into the EcoRI/XhoI sites of the pUAST‐attB vector with a C‐terminal‐fused 3xHA tag by using an In‐Fusion HD Cloning Kit (Clontech) to get the pUAST‐attB_*Fis1*‐3xHA plasmid.

#### 2.9.3 Quantitative real‐time PCR

Total RNA was isolated from fly thoraxes with TRI reagent (Sigma). RNA quantity was evaluated using a microvolume spectrophotometer NanoDrop ND‐2000 (Thermo Fisher). After DNase treatment, first‐strand cDNA synthesis was performed using the SuperScript™ III First‐Strand Synthesis System (Invitrogen). The synthesized cDNA was amplified using a PCR in 10 μl reaction mixtures by using the ViiA 7 Real‐Time PCR System (Applied Biosystems) and the KAPA SYBR^®^ FAST quantitative real‐time PCR (qPCR) Master Mix (2×) Kit (Sigma) with the following procedures: hold stage 95℃ for 20 s; PCR stage 40 cycles of 95℃ for 1 s and 60℃ for 20 s; and melt curve stage 95℃ for 15 s and 60℃ for 1 min. Measurements were taken using $\Delta \Delta C_{t}$ method quantification in triplicates, and relative RNA levels were normalized to rp49 levels.

### 2.10 Colorimetric assay of citrate synthase activity

In accordance with the procedure described by Ping Wei and colleagues (Wei et al., 2020), 10 fly thoraxes were homogenized with 105 μl of extraction buffer (20 mM HEPES [pH = 7.2], 1 mM EDTA, and 0.1% Triton X‐100), with a 5‐μl aliquot for protein content measurement. We added 400 μl of extraction buffer to each sample to increase the total volume to 500 μl and gently mixed. For each reaction, 1 μl of the sample was added to 150 μl of the freshly prepared reaction solution (20 mM Tris–HCl [pH 8.0], 0.1 mM DTNB, 0.3 mM acetyl CoA, and 1 mM oxaloacetic acid). The absorbance (OD) was measured at 412 nm every 10 s for 4 min at 25°C. The reaction rate was calculated using the following equation:

$$\Delta \text{OD}/\Delta T=\left(\text{OD}1‐\text{OD}2\right)/\left(T1‐T2\right).$$

Citrate synthase activity was calculated as follows:

Citrate synthase activity=(reaction rate/protein[mg])×sample dilution.

### 2.11 Hematoxylin and eosin staining

The segment between the thorax and abdomen of 4‐week‐old male flies was fixed in Carnoy's solution (60% ethanol, 30% chloroform, 10% glacial acetic acid) overnight at 4°C. Individual flies were held by their wings with tweezers and glued into a proper orientation on a Petri dish. Five to ten flies of the same genotype were used per test. The following day, samples were embedded in 3% agarose, transferred into 70% ethanol and sent to the pathology core lab of the National Health Research Institutes (NHRI) for hematoxylin and eosin (H&E) staining.

### 2.12 Mitochondria isolation

Mitochondria were purified using a mitochondrial isolation kit (Invitrogen) in accordance with the manufacturer's protocol. In total, 20–100 flies were washed twice with PBS. The PBS was then discarded, and the tissue was ground using a Dounce homogenizer in 400 μl of BSA/Reagent A solution. In addition, 400 μl of Reagent C solution was added, and the tube was inverted several times for mixing (it was not vortexed). Samples were then centrifuged at 700 *g* for 10 min at 4°C. Next, the pellet was discarded and the supernatant was transferred to a new tube. Subsequently, the supernatant cytosol was centrifuged at 12,000 *g* for 15 min at 4°C. After centrifugation, the mitochondrial pellet was isolated from the cytosol, and 500 µl of wash buffer was added to the pellet, which was then suspended by pipetting. Subsequently, the suspension was centrifuged at 12,000 *g* for 5 min and the supernatant was discarded. The mitochondrial pellet was maintained on ice for subsequent experiments.

### 2.13 Proteomic analysis

Proteomic analysis was performed as previously described (Cheung, et al., 2017). Nanoscale liquid chromatography coupled to tandem mass spectrometry (nano LC‐MS/MS) was performed using a mass spectrometer (LTQ‐Orbitrap XL; Thermo Fisher) equipped with a nanoACQUITY UPLC system (Waters). Next, peptide mixtures were loaded onto a 2 cm × 180 μm capillary trap column and then separated in a 75 μm ID × 25‐cm‐long C18 BEH nanoACQUITY column at a flow rate of 300 nl/min, where the mobile phases were A (0.1% formic acid [FA]) and B (0.1% FA/80% acetonitrile [ACN]). A linear gradient of 10%–40% B over 90 min and 40%–85% B over 10 min was used. Mass spectra from full survey scans were acquired on the Orbitrap (*m*/*z* 300–1500). The resolution of the instrument was set to 60,000 at 400 *m*/*z* with an automated gain control (AGC) value of 106. The 10 most intense precursor ions were selected from the MS scan for subsequent collision‐induced dissociation MS/MS scan in an ion trap (AGC target = 7000). For each biological sample, duplicate nano LC‐MS/MS analyses were performed. Two technical replicates were performed for each biological replicate.

### 2.14 ATP concentration measurement

Adult thoraxes were homogenized in water (10 µl/thorax) and centrifuged at 700 *g*. The supernatant was then carefully transferred to new Eppendorf tubes, and nine volumes of 100 mM Tris and 4 mM EDTA (pH 7.55) at boiling temperature were added. The mixture was incubated for 2 min at 100°C and kept on ice after heating. From each volume, 20 μl was obtained to detect the protein concentration by using a Bio‐Rad protein dye assay. At least 50 μl of the remaining samples was then transferred to a 96‐well microplate. Luciferase reagent was added to the samples (sample‐to‐luciferase reagent ratio of 1:1), and the measurement was taken using a microplate reader (425‐155, Hidex Chameleon). The blank was subtracted, and raw sample data were normalized to respective protein concentrations to calculate the relative ATP concentration.

### 2.15 Blue native gel

Pellets of mitochondria isolated from adult flies of the indicated genotypes were suspended at 10 mg/ml in 1× native PAGE sample buffer (Invitrogen) supplemented with protease inhibitor mixture (Sigma) and 2% (w/v) digitonin (Invitrogen). Samples were then immediately centrifuged at 100,000 *g* for 25 min at 4°C. The supernatant was transferred to a new tube, and the native PAGE 5% G‐250 sample additive (Invitrogen) was added. Samples were quickly loaded into a blue native polyacrylamide 3%–12% gradient gel (Invitrogen). After this step, electrophoresis was run in cathode buffer in darkness for 20 min at 150 V, and the condition was then changed to cathode buffer in the light and run for approximately 2 h at 250 V and 4°C. After electrophoresis, gels were fixed in 50% methanol +10% acetic acid for 20 min at room temperature, stained using a Colloidal Blue staining kit (Invitrogen) overnight at room temperature, and destained with deionized water.

### 2.16 Electron microscopy

Heads, abdomens, legs, and wings were excised from 1‐week‐old male flies. The thoraxes were then cut in half and fixed in 0.1 M cacodylate buffer (Sigma; pH 7.2) with 1% glutaraldehyde and 4% paraformaldehyde at 4°C overnight. Tissues were washed three times (15 min each) in 0.1 M cacodylate buffer at 4°C. Tissues were postfixed in OsO_4_/0.1 M cacodylate buffer (pH 7.2) at 4°C for 1 h and washed three times (15 min each) in 0.1 M cacodylate buffer at room temperature (25°C). After washing, the tissues were dehydrated twice in the following increasing concentrations of EtOH: 30%, 50%, and 70% for 10 min; 85% for 20 min; and then 95% and 100% for 20 min at room temperature. Tissues were then soaked twice in propylene oxide (10 min each) at room temperature. Tissue infiltration was conducted at room temperature by using a propylene oxide mixture containing epoxy resin 3:1, 1:1, and 1:3 for 8 h per concentration. Samples were then soaked in resin three times (8 h each) at room temperature and embedded in silicon rubber molds or in gelatin capsules. Finally, tissues were polymerized at 60°C for 24–48 h and sent to Academia Sinica for further sample processing.

### 2.17 Statistical analysis

At least three independent experiments were performed to obtain all results. Values are presented throughout as means ± standard deviations. Statistical data were processed using the GraphPad Prism software and Excel. Analyses utilized either a two‐tailed unpaired Student's *t* test (quantification and comparisons of Western blotting, blue native gel, ATP levels, complex IV activity, 4HNE, uniquitin, ATG8a, muscle vacuole area, and percentage of central myonuclei datasets), Chi‐squared test (quantification of mitochondrial size), Mann–Whitney *U* test (quantification of RCR), ANOVA/Bonferroni's multiple‐comparison test (flight duration experiments), or log‐rank Mantel–Cox test (lifespan experiments). The KEGG pathway enrichment analysis was performed using the ConsensusPathDB Web tool (http://ConsensusPathDB.org). *p* < 0.05 (*), *p* < 0.01 (**), *p* < 0.001 (***), and *p* < 0.0001 (****) were all considered as significant.

## 3 RESULTS

### 3.1 *Fis1* long form expression increased in the thorax during aging

**FIGURE 1:**
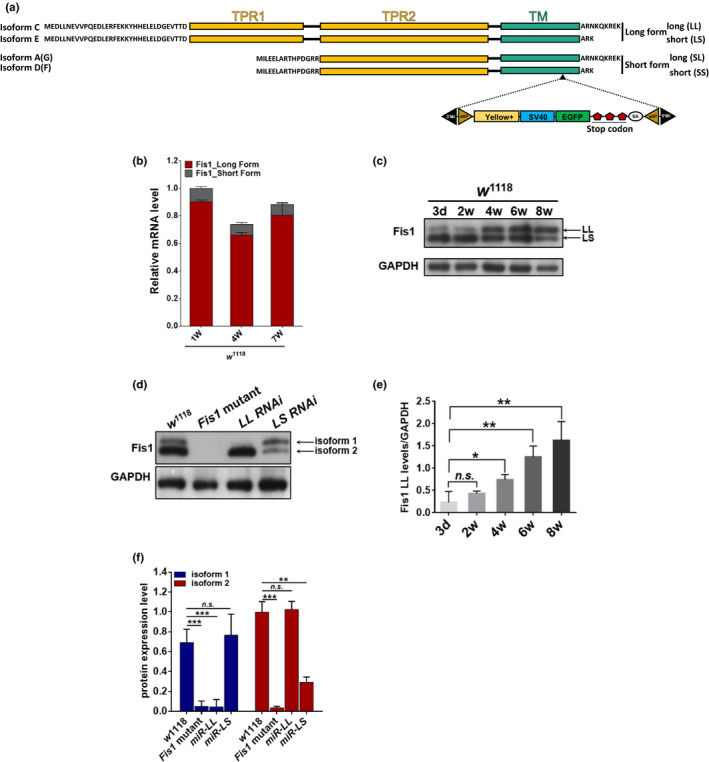
Fis1 LL levels increased with age. (a) Schematic of Fis1 protein isoform structures in a fly. The location of TPR motifs and transmembrane domains (TM) is indicated, and the black triangle indicates the location where the MiMIC cassette was inserted to generate the *Fis1* mutant strain, *Fis1* mutant. (b) qPCR analyses of *Fis1* mRNA levels on weeks 1 and 4 and in *w* ^1118^ male flies. Each group was normalized by the total amount of *Fis1* mRNA level of week‐old *w* ^1118^ males; *n* = 5 replicates with five flies per replicate. (c) Western blot detection and (d) quantification of Fis1 levels in thorax extracts from 4‐week‐old flies of *w* ^1118^, *Fis1* mutant, *mef2*>*UAS*‐*LL*‐*RNAi*, and *mef2*>*UAS*‐*LS*‐*RNAi*. The two detected isoforms are indicated by arrows. The housekeeping protein GAPDH was used as an internal control, and the *w* ^1118^ and *Fis1* mutant lines served as negative controls; *n* = 3 replicates with at least two flies per replicate, **p < 0.01; ***p < 0.001; unpaired *t* test. (e and f) Western blotting detection in flight‐muscle tissue revealed protein levels of endogenous Fis1 LL and LS (indicated by arrows) throughout the lifespan; *n* = 4 replicates with two flies per replicate, *p < 0.05; **p < 0.01; ***p < 0.001; unpaired *t* test

Mitochondrial *Fis1* encodes the mitochondrial outer membrane protein Fis1. The Fis1 gene has been annotated with six transcripts and six polypeptides (four of which are unique) in *D*. *melanogaster*. In this study, we classified the four protein isoforms by the length of their amino acid sequence: short form short (SS), short form long (SL), long form short (LS), and long form long (LL; Figure 1a). All the polypeptides were found to include a single C‐terminal transmembrane domain with cytosolic α‐helixes at the N‐terminus. Unlike the short form group which only contained two α‐helixes, the long form group contained six α‐helixes, which consisted of two tetratricopeptide repeat (TPR) domains (Figure 1a).

To identify the relationship between the expression of the six transcripts of *Fis1* and muscle tissue aging, we examined mRNA levels within the thorax of 1‐, 4‐, and 7‐week‐old flies. qPCR analysis indicated that throughout the *D*. *melanogaster* lifespan, long form transcripts were more highly expressed than short form transcripts (Figures 1b and S1a). Long form transcript levels decreased between the first and fourth weeks, before slightly increasing between the fourth and seventh weeks (Figure 1b). To better understand which long forms were dominant at an endogenous protein level, we generated an antibody for *Fis1* and examined *w* ^1118^ flies through Western blotting. By comparing controls with a MiMIC (Minos‐mediated integration cassette) insertion mutant strain (*Fis1* ^MI10520^, generated by Venken et al., 2011), we observed two protein isoforms (Figure 1c), which were identified as LL and LS through isoform‐specific transgenic targeting and knockdown by RNAi (Figures 1c,d and S1b). To further determine the age‐dependent changes in long form expression levels in *D*. *melanogaster* thoraxes, we examined specimens between 3 days and 7 weeks old. We found that LL expression levels increased with age, whereas LS expression levels remained constant throughout the aging process (Figure 1e,f).

Next, to determine the localization of LL and LS, we generated UAS‐*Fis1*‐LL and UAS‐*Fis1*‐LS lines with HA tags. Driven by muscle‐specific Gal4‐*mef2*, the two transgenic lines were dissected and the inner membrane marker, ATP5a, was discovered to be surrounded by an HA signal, indicating that both of these isoforms localized to the mitochondrial outer membrane (Figure S1c). To further confirm this distribution, we isolated mitochondria from wild‐type flies and examined protein expression through Western blotting. Although LL was localized only to mitochondria, LS was distributed in both the mitochondria and cytoplasm (Figure S1e).

These results suggest that *Fis1* long forms are dominant throughout the *Drosophila* lifespan. The age‐dependent increase in LL suggests a link between *Fis1* and aging.

### 3.2 *Fis1* regulated mitochondrial morphology during aging

The decline in mitochondrial function is a hallmark of aging (López‐Otín et al., 2013). This decline is closely related to changes in the regulation of mitochondrial dynamics, and it may partially determine mitochondrial function (Chan, 2012; Schrepfer & Scorrano, 2016).

**FIGURE 2:**
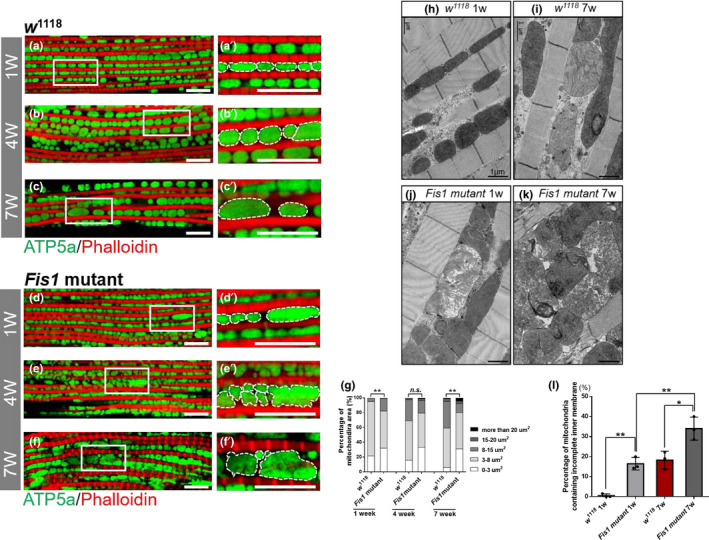
Fis1 regulated mitochondrial morphology in muscle. (a–f) Immunostaining of indirect flight muscles from 1‐week‐old (a and a′), 4‐week‐old (b and b′), and 7‐week‐old (c and c′) *w* ^1118^ flies and 1‐week‐old (d and d′), 4‐week‐old (e and e′), and 7‐week‐old (f and f′) *Fis1* mutant flies. Immunostaining revealed the mitochondrial inner membrane (green channel, anti‐ATP5a) and muscles (red channel, phalloidin/F‐actin); scale bar = 10 μm. (g) Left panel: quantification of mitochondrial area in muscle as shown in (a–b); *n* = 97, 106 (from left to right). **p < 0.01; Chi‐square. Central panel: quantification of mitochondrial area in muscle as shown in (c–d); *n* = 152, 169 from left to right. *p* = 0.1196, Chi‐square. Right panel: quantification of mitochondrial area in muscle as shown in (d–f); *n* = 134, 116 (from left to right); *p* = 0.0019, Chi‐square. (h–i) Representative electron micrographs of mitochondria in 1‐week‐old and 7‐week‐old *w* ^1118^ flight muscles; scale bar = 1 μm, *p* = 0.001. (j–k) Representative electron micrographs of mitochondria in 1‐week‐old and 7‐week‐old *Fis1* mutant flight muscles; scale bar = 1 μm. *p* = 0.0198. (l) Quantification of mitochondria with incomplete inner membrane in (h–k); *n* = 3, every replicate contained one fly. *p < 0.05; **p < 0.01. *p* = 0.001, 0.0097, and 0.0198 from left to right comparison; unpaired Student's *t* test

To investigate whether *Fis1* mutation alters mitochondrial networks in an age‐dependent manner, we monitored mitochondrial morphology in the flight muscles of 1‐, 4‐, and 7‐week‐old *Fis1* mutant flies through staining by using the inner membrane marker ATP5a. Loss of *Fis1* in *D*. *melanogaster* led to enlarged mitochondria in young (1‐week‐old) flies compared with controls of the same age (Fisher's exact test; *p* = 0.0098; Figure 2a,d). Moreover, although mitochondria from control flies exhibited a progressive increase in size over time, we found no difference in mitochondrial size between 4‐week‐old mutant and control flies (*p* = 0.1196; Figure 2b,e), and by 7 weeks, mitochondria were significantly larger in controls than in *Fis1* mutants (*p* = 0.0019; Figure 2c,f).

Next, we examined the mitochondrial ultrastructure of young and aged *Fis1* mutant flies through electron microscopy. We found that the percentage of mitochondria with incomplete cristae increased with age in *w* ^1118^ flies (Figure 2h,i,l). *Fis1* mutant flies possessed larger numbers of impaired mitochondria early in life than did controls, and significantly more impaired mitochondria were observed in later life stages (Figure 2j–l).

These results suggest that loss of *Fis1* increases the incidence of mitochondria with collapsed inner membranes. Because of the appearance of enlarged mitochondria and incomplete cristae, we examined the expression of proteins involved in mitochondrial biogenesis, fission, and fusion in *Fis1* mutants. We investigated the expression of ATP5a (a subunit of ATP synthase), NDUFS3 (a subunit of complex I), porin (a mitochondrial outer membrane component for protein import), and ChChd3 (an inner membrane protein essential for maintaining cristae integrity). ATP5a, ChChd3, and porin exhibited no significant change, whereas NDUFS3 exhibited a significant reduction in expression compared with *w^1118^* flies (Figure S2a,b; *t* test, *p* > 0.05 and *p* < 0.05, respectively). No significant changes in expression level of the fission‐induced protein Drpl or the fusion‐induced protein Marf in *Fis1* mutants compared with *w^1118^* flies were observed (Figure S3; *t* test, *p* > 0.05).

Collectively, the aforementioned data demonstrate that Fis1 regulates mitochondrial morphology without the involvement of mitochondrial dynamic proteins, and loss of *Fis1* impairs the inner membrane structure.

### 3.3 Ablation of *Fis1* impaired mitochondrial inner membrane integrity, leading to defects in mitochondrial function

Mitochondrial dynamics regulate mitochondrial quality and are closely related to mitochondrial function (Lo Verso et al., 2014; Twig et al., 2008; Vainshtein et al., 2014).

According to our analyses, loss of *Fis1* increased the number of mitochondria with incomplete cristae (Figure 2j,k), suggesting a decline in the function of these mitochondria. The main function of mitochondria is to produce ATP through oxidative phosphorylation. To produce ATP, electrons are transferred within the ETC to generate a proton gradient for ATP production. Thus, the integrity of the ETC is crucial for mitochondrial function.

**FIGURE 3:**
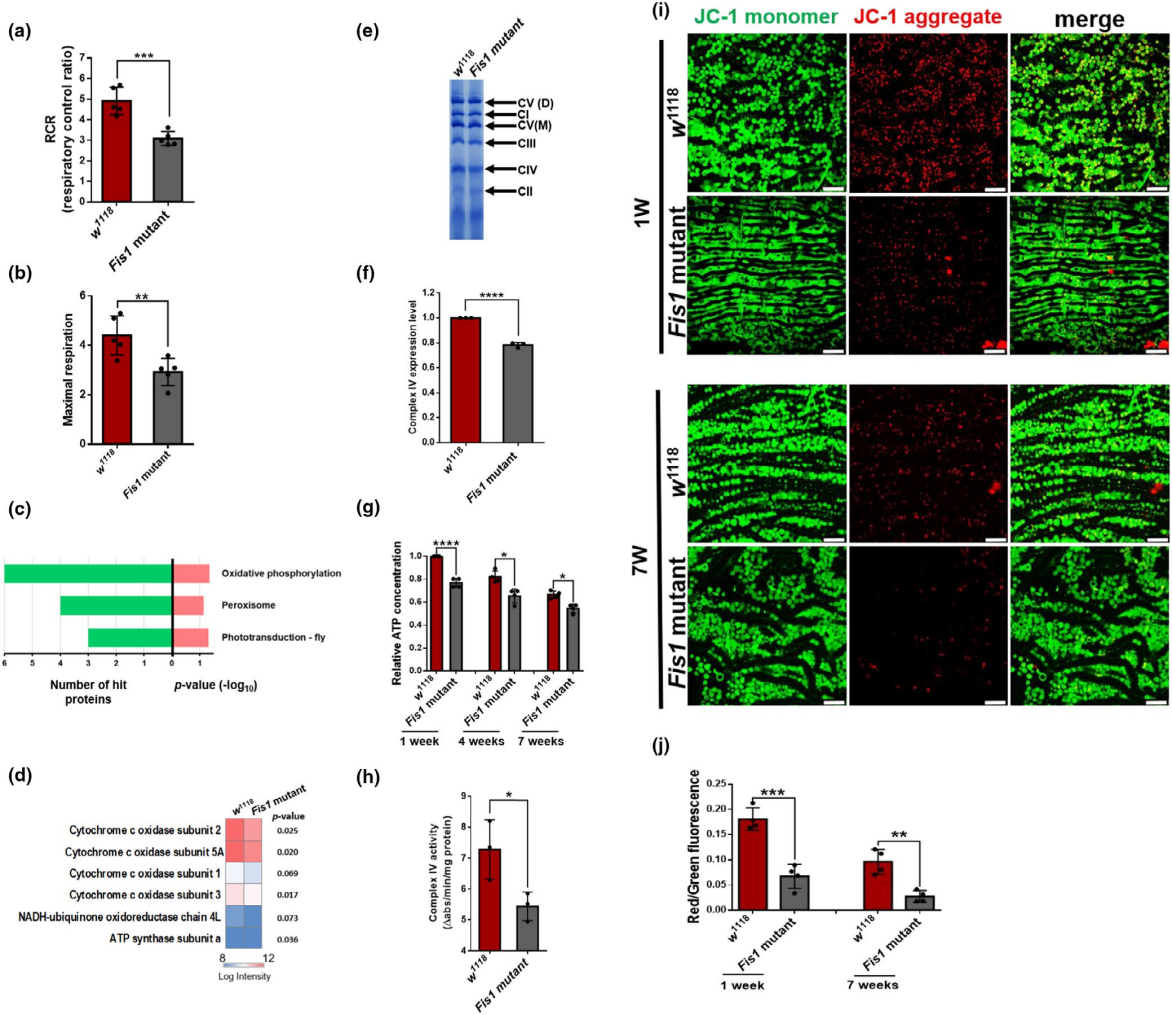
Ablation of *Fis1* impaired mitochondrial inner membrane integrity and led to functional defects in mitochondria. (a–b) RCRs (ADP‐stimulated respiration over basal respiration) and maximal respiration (FCCP‐stimulated over basal respiration) calculations in 6‐week‐old w^1118^ and *Fis1* mutant flies;*p < 0.05, **p < 0.01; Mann–Whitney *U* test. (c) KEGG pathway enrichment analysis of significantly differentially expressed proteins (fold‐change >1.5 or <0.67). Enriched terms are presented with the number of hit proteins (green) or *p* values (pink), respectively. The heatmap depicts the normalized intensities of proteins involved in oxidative phosphorylation function; *n* = 3, every replicate contained at least 100 thoraxes. (d) Heatmap depicting hierarchical mitochondrial proteomics of *w* ^1118^ (left) and *Fis1* mutant flies (right). Red and blue represent the protein intensity from high to low; *p* values are shown as indicated. (e) BN gel of mitochondrial extraction of thoraxes from 4‐week‐old *w* ^1118^ and *Fis1* mutant flies. Each replicate contained at least 100 thoraxes. (f) Quantification of complex IV level as shown in (e); *n* = 3, ****p < 0.0001; Student's *t* test. (h) Activity of citrate synthase in 2‐week‐old *w^1118^* and *Fis1* mutant flies; *n* = 3; each replicate contained ten flies; *p < 0.05; Student's *t* test. (h) Activity of complex IV was examined in 2‐week‐old *w^1118^* and *Fis1* mutant flies; *n* = 3; each replicate contained ten flies; *p < 0.05; Student's *t* test. (i) Immunostaining of indirect flight muscle stained with JC‐1 probe. JC‐1 monomers (green) proportionally accumulated in mitochondria according to the mitochondrial membrane potential. JC‐1 dye tended to aggregate in healthy mitochondria and form JC‐1 aggregates (red). *Fis1* mutant flies had decreased JC‐1 aggregates (red) compared with wild‐type flies during aging. Scale bar: 10 μm. (j) Quantification of JC‐1 aggregate in muscles as shown in (i); *n* = 4, each replicate contained two flies. *p < 0.05 **p < 0.01; ***p < 0.001; unpaired *t* test

We examined mitochondrial respiration by measuring both ADP‐stimulated (RCR) and FCCP‐stimulated (maximal) respiration. Loss of *Fis1* resulted in a lower RCR and maximal respiration activity (Figure 3a,b). This result supports the hypothesis that loss of *Fis1* diminishes respiration chain activity, which may be caused by damage to ETC complexes.

To examine protein levels and the integrity of ETC complexes, we isolated mitochondria specifically from the thorax and subjected the digested proteins to LC‐MS/MS (Figure S4). Among all 13 proteins with upregulated or downregulated expression in *Fis1* mutant flies, those related to oxidative phosphorylation were the most abundant (Figure 3c). All the oxidative phosphorylation‐related proteins were downregulated and contained four subunits of complex IV (Figure 3d). To further determine the content of ETC complexes, we isolated mitochondria from fly thoraxes and examined them using blue native PAGE (BN‐PAGE). Compared with *w* ^1118^ flies, complex IV from the *Fis1* mutant flies appeared to decrease (Figure 3e, quantified in 3f; unpaired *t* test, *p* < 0.0001). The BN‐PAGE results confirmed the decreased expression level of complex IV observed in proteomic data. Because ETC complexes are responsible for producing energy, we tested ATP concentration at different ages and found that *Fis1* depletion resulted in decreased ATP concentration throughout the fly lifespan (unpaired *t* test; *p* < 0.0001 [1w], *p* = 0.0053 [4w], *p* = 0.0169 [7w]; Figure 3g). We also examined the activity of complex IV, which is a crucial catalytic enzyme in the TCA cycle. *Fis1* mutant flies had lower complex IV activity compared with *w* ^1118^ flies, indicating that the TCA cycle is impaired in *Fis1* mutants (Figure 3h). Furthermore, we tested the mitochondrial membrane potential by using JC‐1 and observed a reduced potential in *Fis1* mutant flight muscles (Figure 3i, quantified in 3j; *p* = 0.0004 [1w], *p* = 0.0023 [7w]). Thus, *Fis1* loss results in the ablation of mitochondrial function in muscle tissues.

### 3.4 Depletion of *Fis1* increases ROS production

**FIGURE 4:**
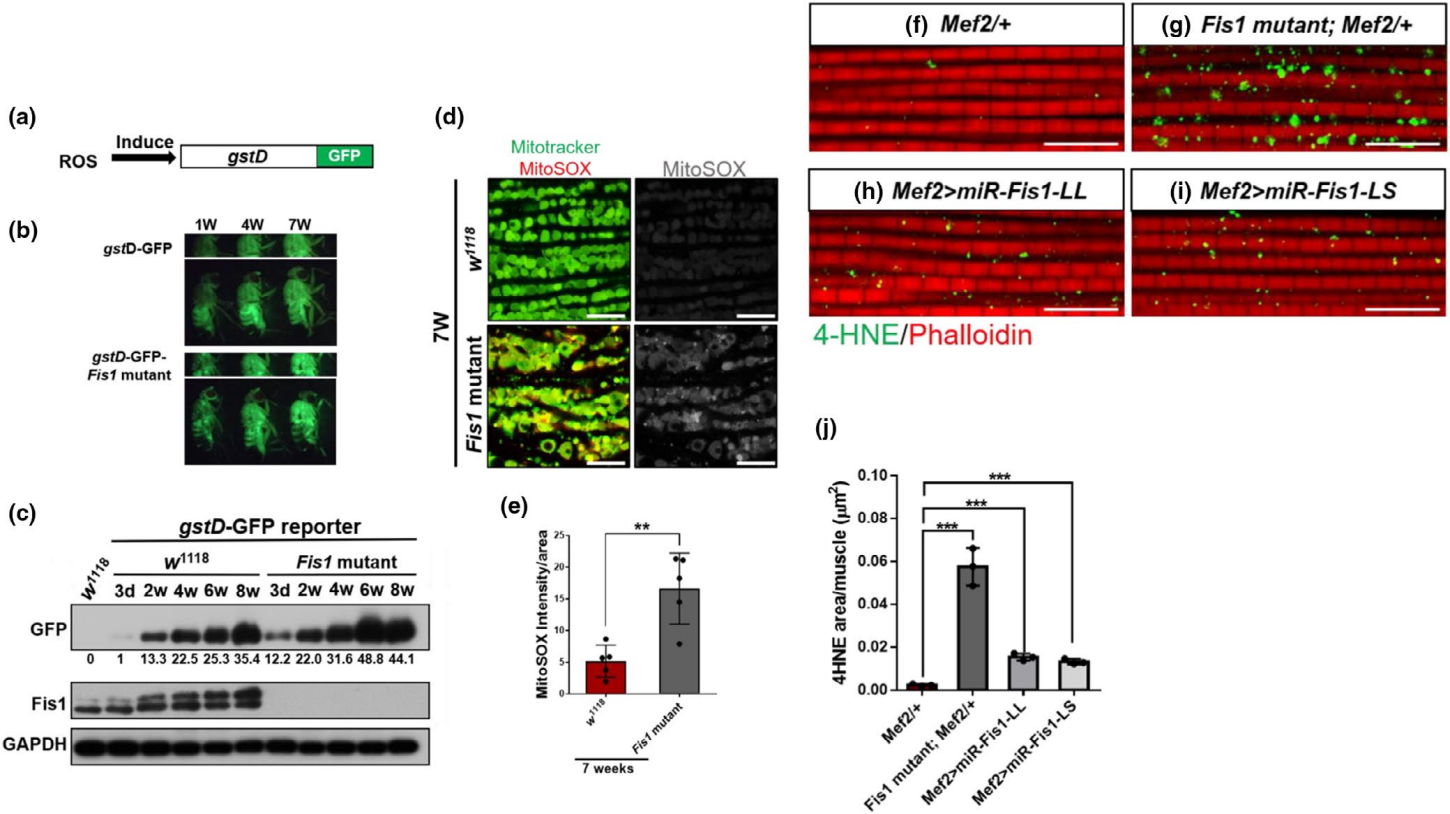
Depletion of *Fis1* increases ROS production and oxidative damages. (a) Structure of the *gstD–GFP* transgene reporter. GFP is produced when *gstD* is induced by ROS. (b) Expression of the transgenic reporter construct *gstD*–GFP was visualized in 1‐, 4‐, and 7‐week‐old flies (from left to right) through fluorescence stereomicroscopy. Transcription of the *gst*D enhancer was potently induced in the gut and in other tissues using oxidants. (c) Western blot analysis of GFP expression levels in thoracic tissues of homozygous *gstD–*GFP reporter alone or *gstD–*GFP–Fis1 recombined reporter upon various ages. Each group of GFP intensity was compared with the GFP intensity of 3‐day‐old *gstD–*GFP reporter. The relative intensity was calculated and labeled. (d) Staining of indirect flight muscles from 7‐week‐old *w* ^1118^ and *Fis1* mutant males, showing mitochondria (green channel, Mitotracker green staining) and levels of superoxide radicals (red channel, staining with MitoSOX reagent); scale bar = 10 μm. (e) Quantified ratio of mitoSOX signal to mitochondrial area as depicted in (d); unpaired *t* test, *n* = 4, each replicate contained two flies. **p < 0.01; ***p < 0.001. (f–i) Immunostaining of indirect flight muscles from 4‐week‐old *Mef2 control*, *Fis1* mutant, *mef2*>*miR*‐*LL*, and *mef2*>*miR*‐*LS* flies, showing 4‐HNE aggregates (green channel) and phalloidin (red channel); scale bar = 10 μm. (j) Quantification of accumulated 4‐HNE aggregates in muscle as depicted in (f–i). The area of aggregated 4‐HNE was normalized by the total mitochondria area. Unpaired *t* test, *n* = 3, each replicate contained two flies. ***p < 0.001; unpaired *t* test

Mitochondria are the main source of ROS (Ábrigo et al., 2018; Balaban et al., 2005; Cadenas & Davies, 2000; Starkov & Fiskum, 2003). A study reported that the mutation of complex IV led to ROS leakage in aged mice (Reichart et al., 2019). We assumed that a lack of *Fis1* would increase ROS levels in *Fis1* mutant flies; therefore, we expressed the ROS reporter *gstD* with a GFP tag in the fly thorax (Sykiotis & Bohmann, 2008; Figure 4a), which allowed us to monitor the basal level of GFP in flies. In adult flies carrying the *gstD*–GFP reporter, GFP activity can be used to monitor the production of ROS at basal conditions after subtracting the baseline GFP signal from the abdomen (Figure 4b). By using Western blotting, we found that aged flies exhibited an increase in GFP levels. In addition, *Fis1* mutant flies produced a greater amount of ROS than did wild‐type flies at all ages tested (Figure 4c), suggesting that the unusually high level of ROS resulted from loss of *Fis1*. Next, to confirm whether the elevated ROS originated from mitochondria, we stained 7‐week‐old flight muscles with mitochondrial ROS indicator (mitoSOX) and found significantly increased mitochondrial ROS in *Fis1* mutant flies (Figure 4d, quantified in 4e; *p *< 0.001). Excess ROS provokes the oxidation of phospholipids in mitochondrial membranes and forms lipid peroxidative products (Yin et al., 2011). Among these products, 4‐hydroxy‐2‐nonenals (4‐HNE) is one of the most bioactive and representative lipid alkenals (Benedetti et al., 1980). When stained using the 4‐HNE antibody, *Fis1* mutant flies also exhibited increased 4‐HNE signals within muscle mitochondria, indicating increased oxidative damage (Figure 4f,g; quantification in 4g; *p *< 0.0001). Specifically, by using *mef2*>*Fis1*‐*LL RNAi* and *ef2*>*Fis1*‐*LS RNAi* flies, we verified that the knockdown of Fis1‐LL or Fis1‐LS resulted in increased oxidative damage in the muscles of mutant flies (Figure 4f–i, quantified in 4j). We also found that the ectopic expression of Fis1‐LL or Fis1‐LS suppressed the upregulation of 4‐HNE in *Fis1* mutants (Figure S5d–g). Collectively, loss of both long forms of Fis1 increased ROS levels and the amount of oxidative lipids in the thoraxes of flies, which consequently increased oxidative damage.

### 3.5 Depletion of *Fis1* leads to remarkable ubiquitinated protein aggregation during aging

Accumulated ubiquitinated protein in muscle is representative of aging, and oxidative stress is known to play a role in regulating proteostasis (Fernando et al., 2019).

However, the role of increased ROS in disrupting proteolytic homeostasis in muscle remains unknown. In this study, we examined whether mitochondrial ROS, which is caused by *Fis1* loss, causes an imbalance in proteostasis in muscle.

**FIGURE 5:**
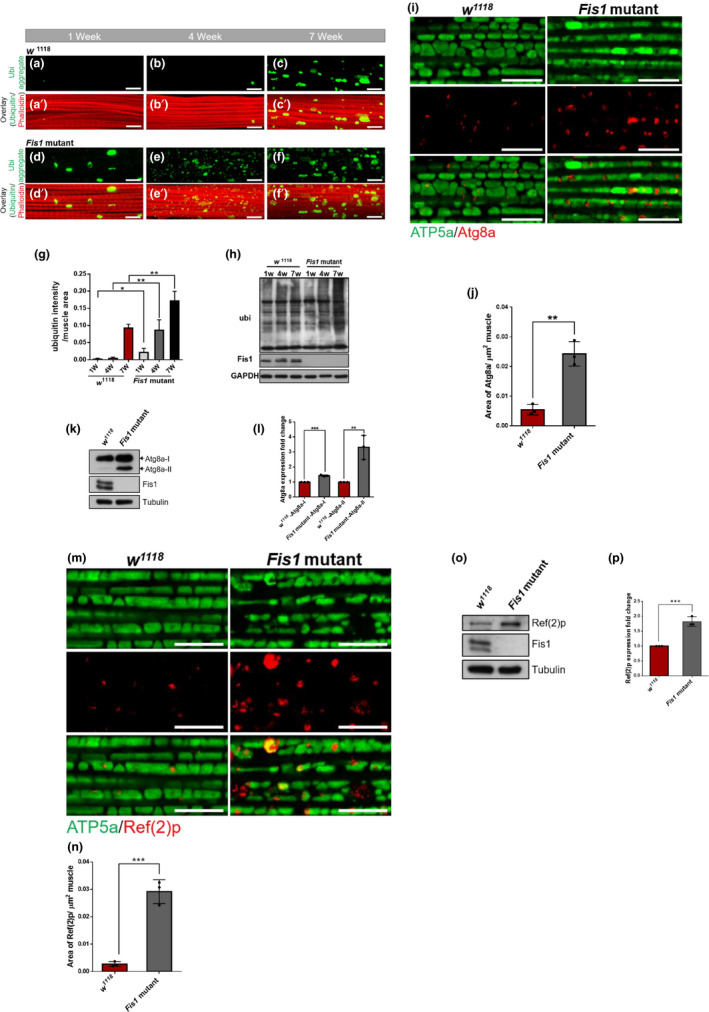
Protein degradation pathways were impaired with Fis1 inhibition in adult muscles. (a–c′) Immunostaining of indirect flight muscles from 1‐week‐old (a and a′), 4‐week‐old (b and b′), and 7‐week‐old (c and c′) *w* ^1118^ flies, showing polyubiquitinated aggregates (green channel, anti‐FK2) and muscles (red channel, phalloidin/F‐actin); scale bar = 10 μm. (d–f′) Immunostaining of indirect flight muscles from 1‐week‐old (d and d′), 4‐week‐old (e and e′), and 7‐week‐old (f and f′) *Fis1* mutant flies, showing polyubiquitinated aggregates (green channel, anti‐FK2) and muscles (red channel, phalloidin/F‐actin); scale bar = 10 μm. (g) Quantification of polyubiquitin aggregates in muscle as depicted in (a–f); *n* = 3, each replicate contained two flies. *p < 0.05; **p < 0.01; unpaired *t* test. (h) Western blot detection and densitometry of total ubiquitin‐conjugated proteins between *w* ^1118^ and *Fis1* mutant flies between 1 and 7 weeks old. (i) Immunostaining of indirect flight muscles from 2‐week‐old *w* ^1118^ and *Fis1* mutant flies, showing mitochondria (green channel, anti‐ATP5a) and Atg8a aggregates (red channel, anti‐Atg8a); scale bar = 10 μm. (j) Quantification of Atg8a aggregates as depicted in (i); *n* = 3, each replicate contained two flies. **p < 0.01; unpaired *t* test. (k) Atg8a‐II was accumulated in 2‐week‐old Fis1 mutant. (l) Quantification of Atg8a‐II as depicted in (K); *n* = 3, each replicate contained two flies. **p < 0.01; unpaired *t* test. (m) Immunostaining of indirect flight muscles from 2‐week‐old *w* ^1118^ and *Fis1* mutant flies, showing mitochondria (green channel, anti‐ATP5a) and Ref(2)p aggregates (red channel, anti‐Ref(2)p); scale bar = 10 μm. (n) Quantification of Ref(2)p aggregates as shown in (m); *n* = 3, each replicate contained two flies. ***p < 0.001; unpaired *t* test. (o) Ref(2)p was accumulated in 2‐week‐old Fis1 mutants. (p) Quantification of Ref(2)p as shown in (O); *n* = 3, each replicate contained two flies. ***p < 0.001; unpaired *t* test

We first examined the accumulation of ubiquitinated protein within flight muscles through immunofluorescence microscopy and found a predominant accumulation of ubiquitinated protein in week‐old *Fis1* mutant flies compared with *w* ^1118^ flies (Figure 5a,d and a′,d′). Furthermore, *Fis1* loss not only caused premature ubiquitin accumulation but also highly accelerated the accumulation (Figure 5a–f, quantified in 5g). These results were also confirmed by Western blotting (Figure 5h), suggesting that *Fis1* participates in the protein degradation system throughout the fly lifespan and in the accumulation of ubiquitinated protein in early age. Therefore, loss of *Fis1* accelerated the accumulation of ubiquitinated proteins in muscle during aging.

Studies have described how the muscle‐specific inhibited mitochondrial fission protein Drp1 leads to a reduction of mitochondrial function and elevation of impaired autophagy markers in mammal muscle, which results in an atrophy phenotype (Dulac et al., 2020; Favaro et al., 2019).

To further clarify whether a lack of *Fis1* impaired autophagy within the muscle cells in flies, we probed muscles with the steady‐state autophagy marker Atg8a to visualize the endogenous autophagosome. We found that 2‐week‐old *Fis1* mutant flies accumulated Atg8a protein (Figure 5i, quantified in 5j). The increased aggregation of Atg8a could also be observed in Western blotting (Figure 5k, quantified in 5l). Furthermore, similar to the accumulation of ubiquitinated protein, the level of aggregated Ref(2)p in 2‐week‐old mutant flies significantly increased compared with *w* ^1118^ flies (Figure 5m, quantified in 5n). The increased aggregation of Ref(2)p could also be observed in Western blotting (Figure 5o, quantified in 5p). These data indicate that loss of *Fis1* impairs the proteostasis mechanism in fly muscles from a young age.

### 3.6 *Fis1* deletion causes myopathy in indirect flight muscle and led to exercise intolerance

Changes in proteostasis can accelerate aging (Labbadia & Morimoto, 2015). Without an appropriate clearance system for damaged proteins, increased cytotoxicity may occur and the lifespan may be shortened (Kevei & Hoppe, 2014).

**FIGURE 6:**
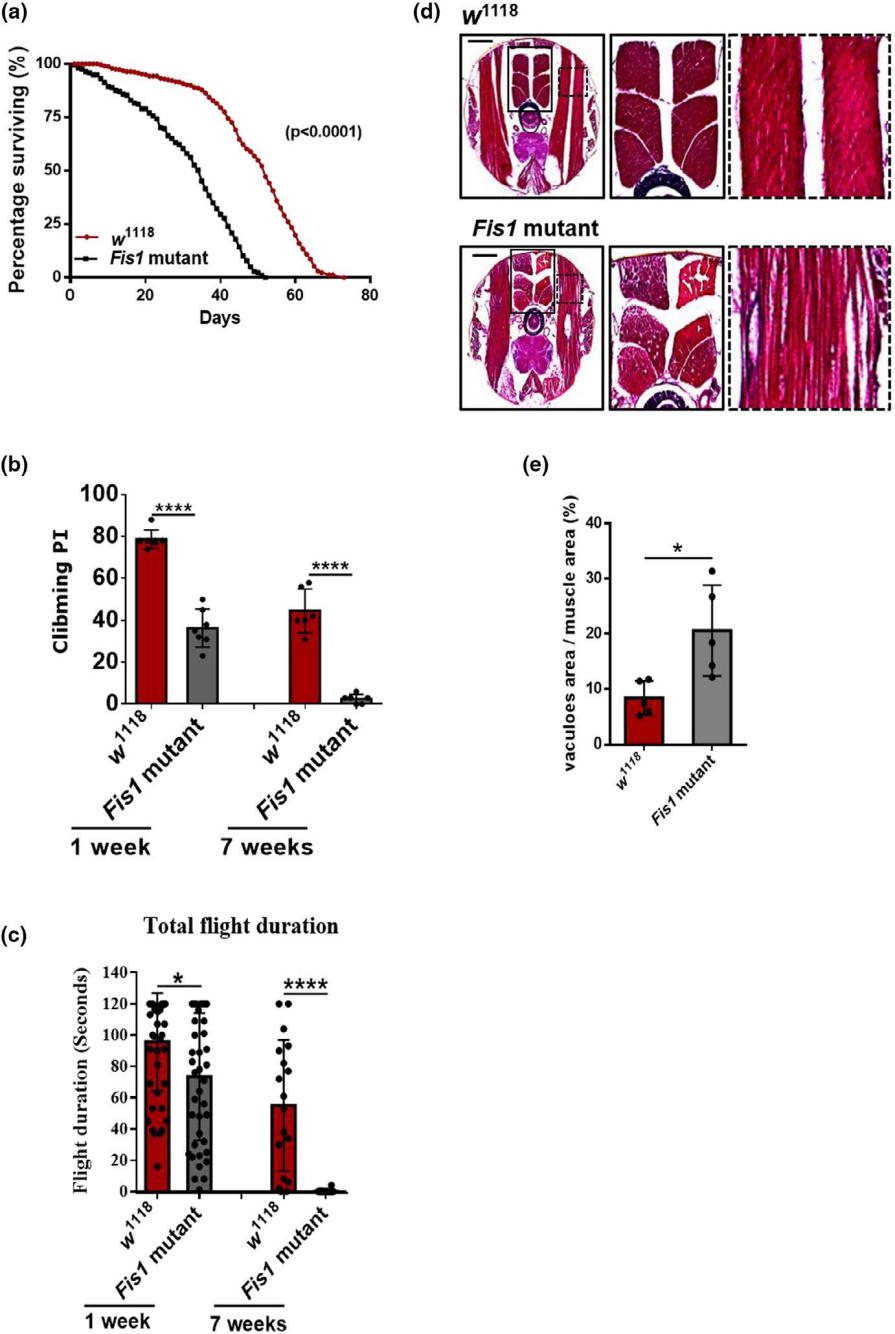
*Fis1* deletion caused myopathy in indirect flight muscles and led to exercise intolerance. (a) The survival curve of *w* ^1118^ and Fis1 mutants indicates that Fis1 deletion resulted in lethality after eclosion. *w* ^1118^ flies, *n* = 347; Fis1 mutants, *n* = 219. ****p < 0.0001; log‐rank (Mantel–Cox) test. (b) Compounded climbing PI of 1 and 7‐week‐old *w* ^1118^ and mutant flies. The PIs of Fis1 mutants were analyzed and compared with those of *Fis1 w* ^1118^ flies; *n* = 6, each replicate contained at least 90 flies. ****p < 0.0001; Student's *t* test. (c) Total flight duration was significantly reduced in animals with knockdown of Fis1 compared with controls at 1 and 7 weeks old. From left to right, *n* = 47, 42, 18, 20. *p < 0.05; ****p < 0.0001; ANOVA/Bonferroni's multiple‐comparisons test. (d) H&E staining of flight muscle revealed that 4‐week‐old Fis1 mutant flies lost muscle fiber integrity compared with controls. (e) Quantified ratio of vacuole area to muscle area as shown in (d); *n* = 5, *p < 0.05; Student's *t* test

We found that *Fis1* mutant flies exhibited a reduced median lifespan (37 days) by almost one‐third compared with wild‐type flies (55 days; Figure 6a; *p* < 0.0001). Similarly, the use of *mef2* gal4 to drive *UAS*‐*Fis1 LL* or *LS* RNAi targeting led to a shortened lifespan (Figure S6; *p* < 0.0001). Because age‐related diseases are often accompanied by impaired mobility, we tested the climbing ability and flight maintenance capability of both young and old flies. Both tests demonstrated that flies lacking *Fis1* exhibited significantly decreased mobility at all ages, which worsened with age (Figure 6b,c).

In neurodegenerative conditions such as AD and Parkinson's disease, proteostasis dysfunction leads to the aberrant accumulation of proteins, causing neuron death (Sweeney et al. 2017). This disease process can also be observed in muscles, leading to the degeneration of muscle cells (Fernando et al., 2019). The evidence of accumulated ubiquitin within muscles and declined mobility led us to investigate the possibility of muscle degeneration in *Fis1* mutant flies. To test whether these mobility defects resulted from muscle degeneration, we performed H&E staining on *Fis1* mutant flies and found severe muscle degeneration; specifically, the impaired indirect flight muscles were packed in a disorderly manner and vascular areas were visible (Figure 6d, quantified in 6e).

In addition, the number of centrally nucleated myofibers has been used as a marker to reflect previous cycles of muscle degeneration and regeneration (Dulac et al., 2020; Favaro et al., 2019; Sakellariou et al., 2011). *Drp1* mutants exhibited accelerated muscle degeneration with the appearance of centrally localized myonuclei (Dulac et al., 2020; Favaro et al., 2019). To further examine *Fis1*‐induced muscle degeneration, we examined the location of myonuclei in *Fis1* mutants.

By using phalloidin to indicate the muscle area and DAPI as a nucleus marker, we found a higher ratio of centrally located myonuclei in the aged control flies compared with the young control flies (Figure S7a,b, quantified in e). However, aged *Fis1* mutant flies had a higher ratio of centrally nucleated myofibers than did the aged control flies (Figure S7c,d, quantified in e), indicating that loss of *Fis1* accelerates muscle degeneration. Together, these findings indicate that loss of *Fis1* causes an imbalance in mitochondrial proteostasis within muscle, affecting the health and lifespan of *Drosophila*.

## 4 DISCUSSION

Results from numerous model systems have suggested that aging is linked to alterations in the regulation of mitochondrial dynamics (Sharma et al., 2019). However, the underlying mechanisms that modulate these changes and the causal relationships between altered mitochondrial dynamics and age‐related health decline remain unclear. In this study, we addressed whether *Fis1*, an outer membrane fission factor in yeast, could modulate tissue and organismal aging in *Drosophila*. We first discovered that the mRNA of long form *Fis1* transcripts was dominant throughout the *Drosophila* lifespan. In addition, we found that the protein expression levels of LL increased with age, whereas equivalent levels of LS had a mild effect.

We further found that loss of Fis1‐LL or Fis1‐LS in muscle resulted in elevated ROS levels (Figure 4f–j), and their ectopic expression led to the suppression of oxidative stress (Figure S5). These results suggest that both Fis1‐LL and Fis1‐LS have similar functions in influencing the production of ROS and oxidative lipids. Although LL and LS are similar in protein sequence and structure, their C‐terminal tails differ slightly; therefore, LL possesses greater similarity to human *Fis1*. Another study reported that the transmembrane domain, which is located at the C‐terminus, is crucial for human *Fis1* to function as a regulator of mitochondrial dynamics (Yu et al., 2019). The differences in C‐terminal structure between LL and LS may guide them to different locations as well as cause them to have different functions.

Muscle proteomic results in humans indicated that the expression of the mitochondrial fission component dynamin 2 increases with age (Murgia et al., 2017). *Fis1* and S‐nitrosylated Drp1 (SNO‐Drp1) significantly increased in brain tissues and skin fibroblasts in patients with AD (Wang et al., 2012).

Of the two major isoforms of *Fis1* in *D*. *melanogaster*, LL possesses a greater similarity to human *Fis1*. The increased LL levels suggest that LL has potential for use as a biomarker of aging or as a therapeutic target against aging or age‐related diseases. Future work can focus on whether LL possesses specific functions in aging and whether these functions differ among other isoforms.

Inducing mitochondrial fission is known to rescue deterioration in PINK1 and Parkin mutant flies (Deng et al., 2008; Yang et al., 2008), supporting the hypothesis that mitochondrial fission can facilitate mitophagy (Mao & Klionsky, 2013; Twig et al., 2008). However, little is known about the relationships among mitochondrial dynamics, mitophagy, and aging. *Fis1* is involved in recruiting Dnm1p (the yeast Drp1 homolog) to mitochondria through interaction with Mdv1p and Caf4p, and it is involved in promoting mitochondrial fission processes in yeast (Hoppins et al., 2007). Given the absence of Mdv1p and Caf4p in metazoans, the actual role of *Fis1* in the mitochondrial fission process remains unclear (Hoppins et al., 2007; Okamoto & Shaw, 2005). Some studies have suggested that *Fis1* participates in fission processes (James et al., 2003; Shen et al., 2014; Stojanovski et al., 2004) or inhibits fusion factors (Yu et al., 2019), whereas others have suggested that *Fis1* is dispensable to fission processes or has only mild effects on mitochondrial morphology when mutated or overexpressed (Shen et al., 2014; Suzuki et al., 2003).

In this study, we examined the mitochondrial morphology of *Fis1* mutant flies at different ages (1, 4, and 7 weeks) and found that loss of *Fis1* led to enlarged mitochondria at young (1 week) and old (7 weeks) ages. However, the mutation of *Fis1* had a minor effect on mitochondrial morphology at 4 weeks.

Loss of *Fis1* increased the number of enlarged mitochondria with incomplete cristae during aging, suggesting that Fis1 regulates mitochondrial morphology in an age‐dependent manner. Furthermore, mitochondrial size and Fis1‐LL protein expression increased with age, and the ectopic expression of Fis1‐LL not only induced smaller mitochondria (Figure S1c,d) but also reduced oxidative damage (Figure S5a–c). We propose that Fis1 may have a protective function during aging; however, the increased expression of Fis1‐LL with age is insufficient for fully suppressing age‐related mitochondrial impairment. We believe that Fis1‐LL plays a role in maintaining proteostasis and preventing the overenlargement of mitochondria; therefore, loss of *Fis1* should cause mitochondrial enlargement and incomplete cristae during aging (Figure 2). However, further investigations are required to clarify the detailed mechanism.

Mitochondrial dynamics (fission/fusion) and mitophagy are closely related mechanisms for maintaining a healthy mitochondrial pool and eliminating damaged mitochondria (Ashrafi & Schwarz, 2013; Lo Verso et al., 2014; Twig et al., 2008; Twig & Shirihai, 2011; Vainshtein et al., 2014). In this study, we found that Fis1 plays a protective role in proteostasis. *Fis1* mutation compromised the integrity of the mitochondrial inner membrane in young flies and was accompanied by defects in complex IV. A study suggested that the mutation of complex IV in a mouse model reduced lifespans with increased ROS production as well as downregulation of *Fis1* (Reichart et al., 2019). However, elevated ROS levels in aged *w* ^1118^ flies were also observed; this observation is crucial. Oxidative stress is defined as an imbalance between the production of ROS and the antioxidant defense systems (Luceri et al., 2018). The accumulating ROS attack DNA, induce DNA damage, and disrupt the antioxidant defense systems (Peng et al., 2014), causing age‐related aggregation of ROS in control strains. Our study suggests that loss of *Fis1* produces an imbalance in oxidative stress through defects in complex IV, which potentially mediates mitochondrial ROS production.

Furthermore, defects within the ETC not only increase ROS levels but also downregulate ATP concentration. Our findings indicate that mitochondria in *Fis1* mutant flies exhibit reduced membrane potentials, suggesting a decline in mitochondrial function, which occurs early in the aging process. Because this cumulative damage is toxic to cells, it is crucial to clear dysfunctional mitochondria. *Fis1* was reported to regulate PINK1‐independent mitophagy in humans (Xian et al., 2019), and mutation of *Fis1* was found to induce abnormal mitophagy in a mouse model (Zhang et al., 2019). We observed a dramatic accumulation of ubiquitinated protein aggregates in the muscles of young *Fis1* mutant flies. These aggregates continuously accumulated with age, and eventually mutant muscle fibers were almost completely filled with protein aggregates. This accumulation is a striking phenotype of aging. Furthermore, an endogenous autophagic marker, Atg8a, aggregated during early life stages. *Fis1* may thus be a key protein linking mitochondrial function and mitophagy during aging.

Several AD animal models and AD patient brains have exhibited evidence of reduced ATP concentration, diminished complex IV activity, and enhanced oxidative stress compared with controls (Hauptmann et al., 2009; Reddy, 2009; Xie et al., 2013). *Fis1* has also been reported to be a potential biomarker of AD because its expression is increased in the peripheral blood lymphocytes of patients with AD (Wang et al., 2012). Moreover, increased levels of Drp1–*Fis1* interaction, which promotes mitochondrial fission, have been discovered in fibroblasts derived from patients with AD (Joshi et al., 2018). A study also reported that the C9ORF72 gene, the mutation of which causes amyotrophic lateral sclerosis, has a strong and lethal genetic interaction with *Fis1* (Chai et al., 2020). These studies implicate *Fis1* as a causal factor of the aforementioned diseases. Finally, our findings reveal how loss of *Fis1* can lead to a decline in mitochondria and eventual acceleration of the aging process.

## CONFLICT OF INTERESTS

The authors declare no competing interests.

## AUTHOR CONTRIBUTIONS

T.‐T.L., P.‐L.C., J.‐C.L., S.V.S., E.Z, and C.‐H.C. conceived and designed experiments; J.‐C.L. designed and assembled plasmids; T.‐T.L., P.‐L.C., J.‐C.L., Y.‐W.C., and S.V.S. performed experiments; T.‐T.L., P.‐L.C., Y.‐W.C., H.‐F.J., M.P.S., and C.‐H.C. analyzed data; and T.‐T.L., P.‐L.C., J.‐C.L., M.P.S., Y.‐W.C., J.‐M.Y., H.‐D.W., and C.‐H.C. wrote the paper.

## Supporting information

Fig S1‐S7

## Data Availability

The data that support the findings of this study are available from the corresponding author upon reasonable request.
