# Supplementary material for: Loss of Fis1 impairs proteostasis during skeletal muscle aging in Drosophila
Source: Aging Cell. 2021 Jun 1;20(6):e13379. doi: 10.1111/acel.13379 (PMC8208795; doi:10.1111/acel.13379)
Supplement: Supplementary file 1 — Fig S1‐S7 [file ACEL-20-e13379-s001.pdf]

Supplemental Information

Figure S1

S1A

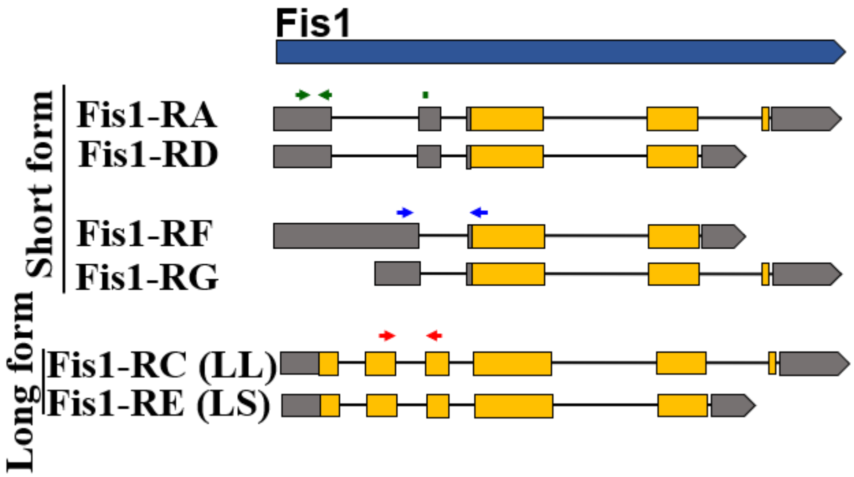

S1B

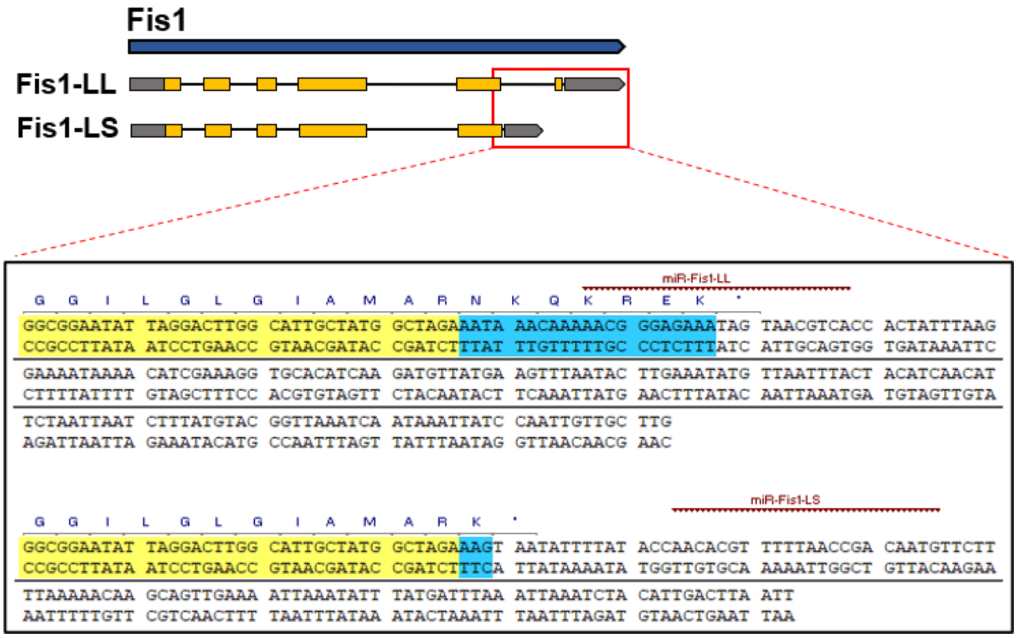

Figure S1

S1C

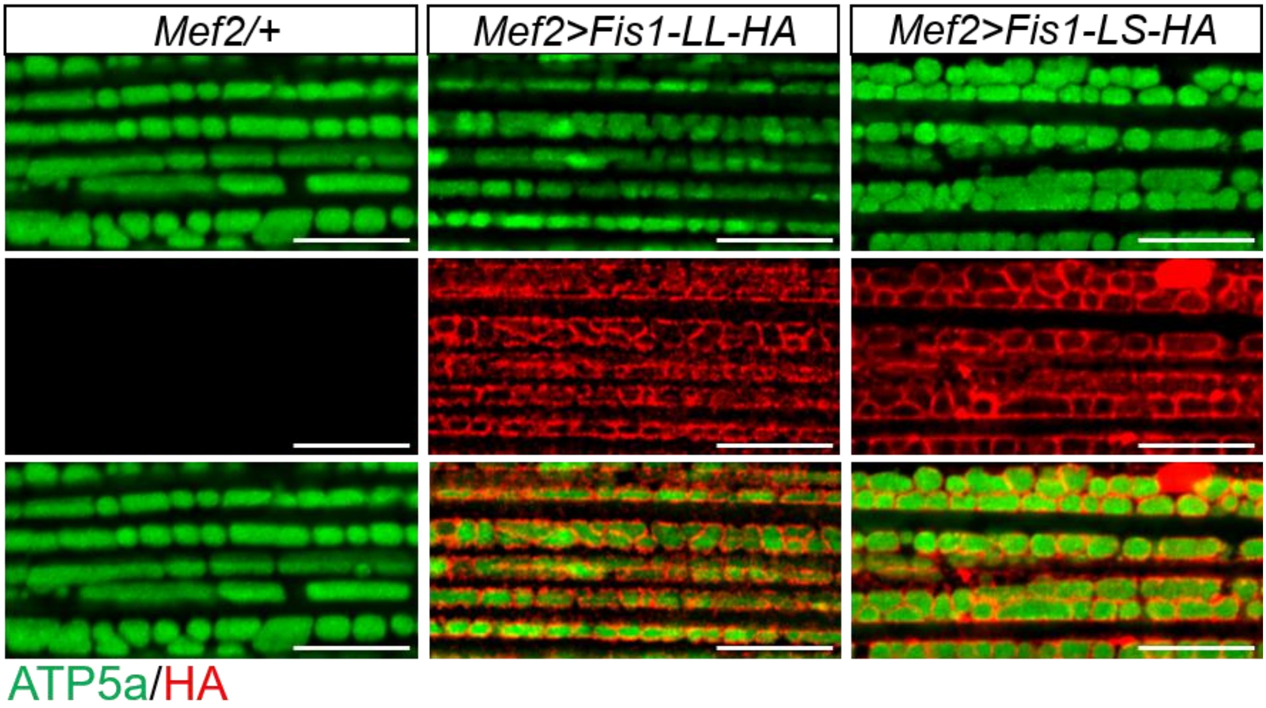

S1D

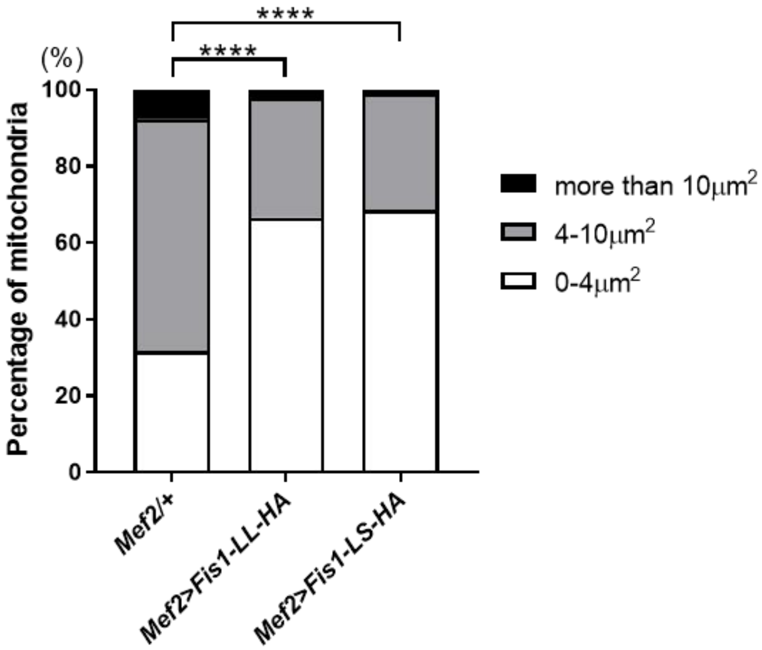

Figure S1

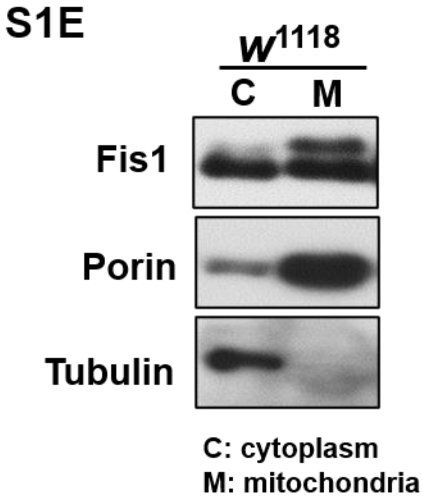

Figure S2

S2A

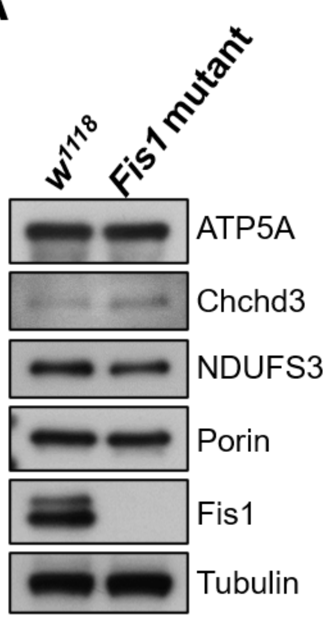

S2B

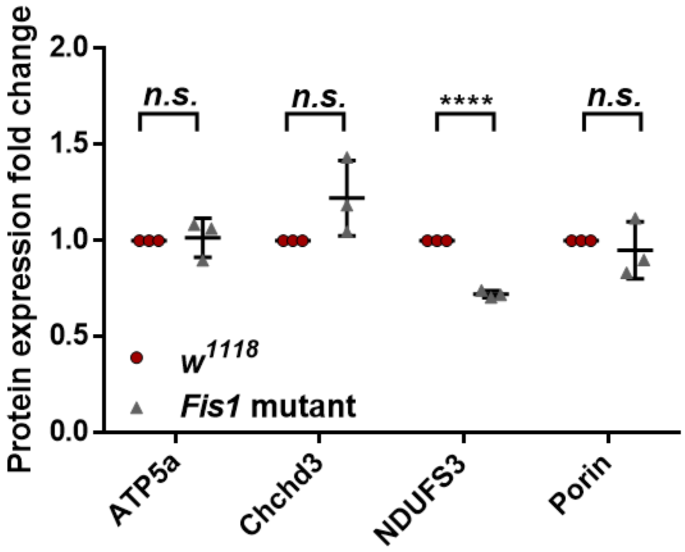

Figure S3

S3

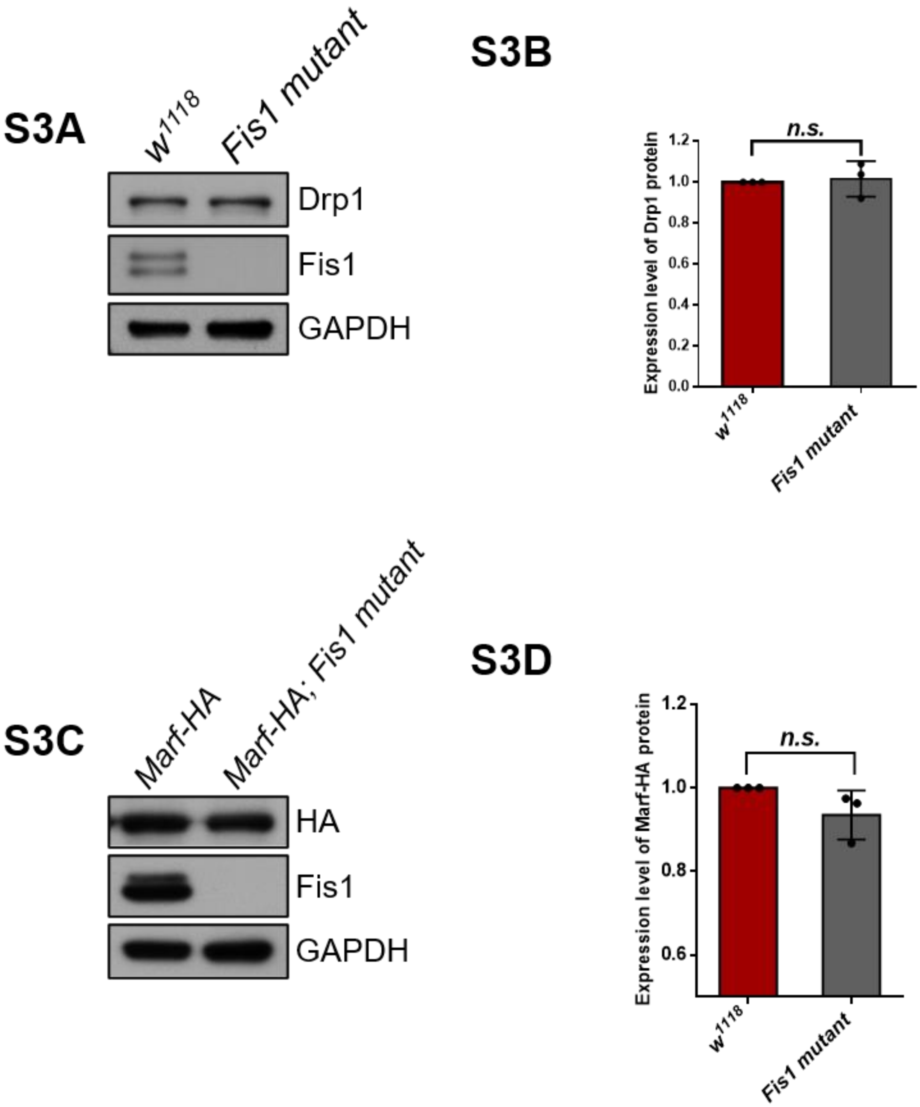
S3A
w1118
Fis1 mutant
Drp1
Fis1
GAPDH
S3B
n.s.
Expression level of Drp1 protein
1.2
1.0
0.8
0.6
0.4
0.2
0.0
w1118
Fis1 mutant
S3C
Marf-HA
Marf-HA; Fis1 mutant
HA
Fis1
GAPDH
S3D
n.s.
Expression level of Marf-HA protein
1.2
1.0
0.8
0.6
w1118
Fis1 mutant

Figure S4

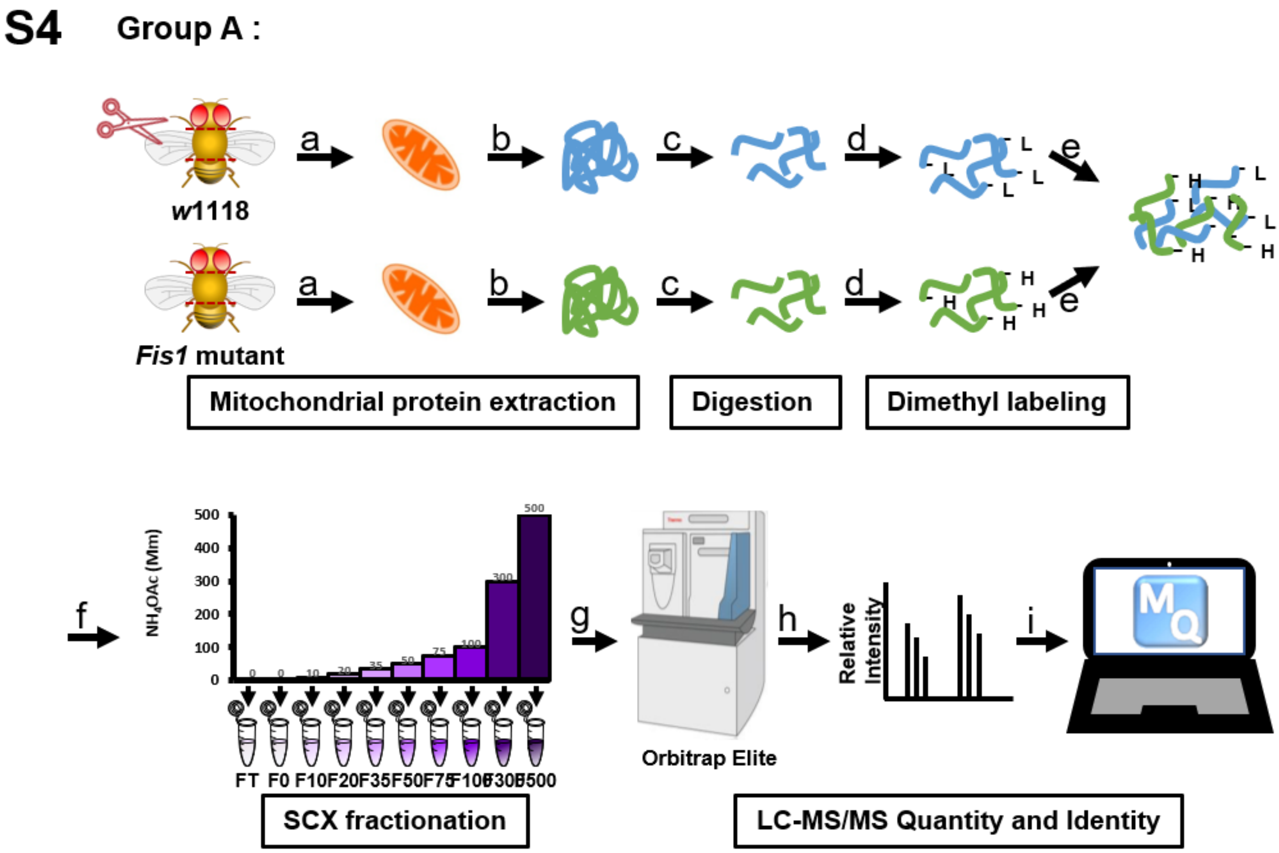
S4
Group A :
w1118
Fis1 mutant
a
b
c
d
e
L
H
Mitochondrial protein extraction
Digestion
Dimethyl labeling
f
NH4OAc (Mm)
500
400
300
200
100
0
10
20
35
50
75
100
FT F0 F10 F20 F35 F50 F75 F100 F300 F500
SCX fractionation
g
Orbitrap Elite
h
Relative Intensity
i
MQ
LC-MS/MS Quantity and Identity

Figure S5

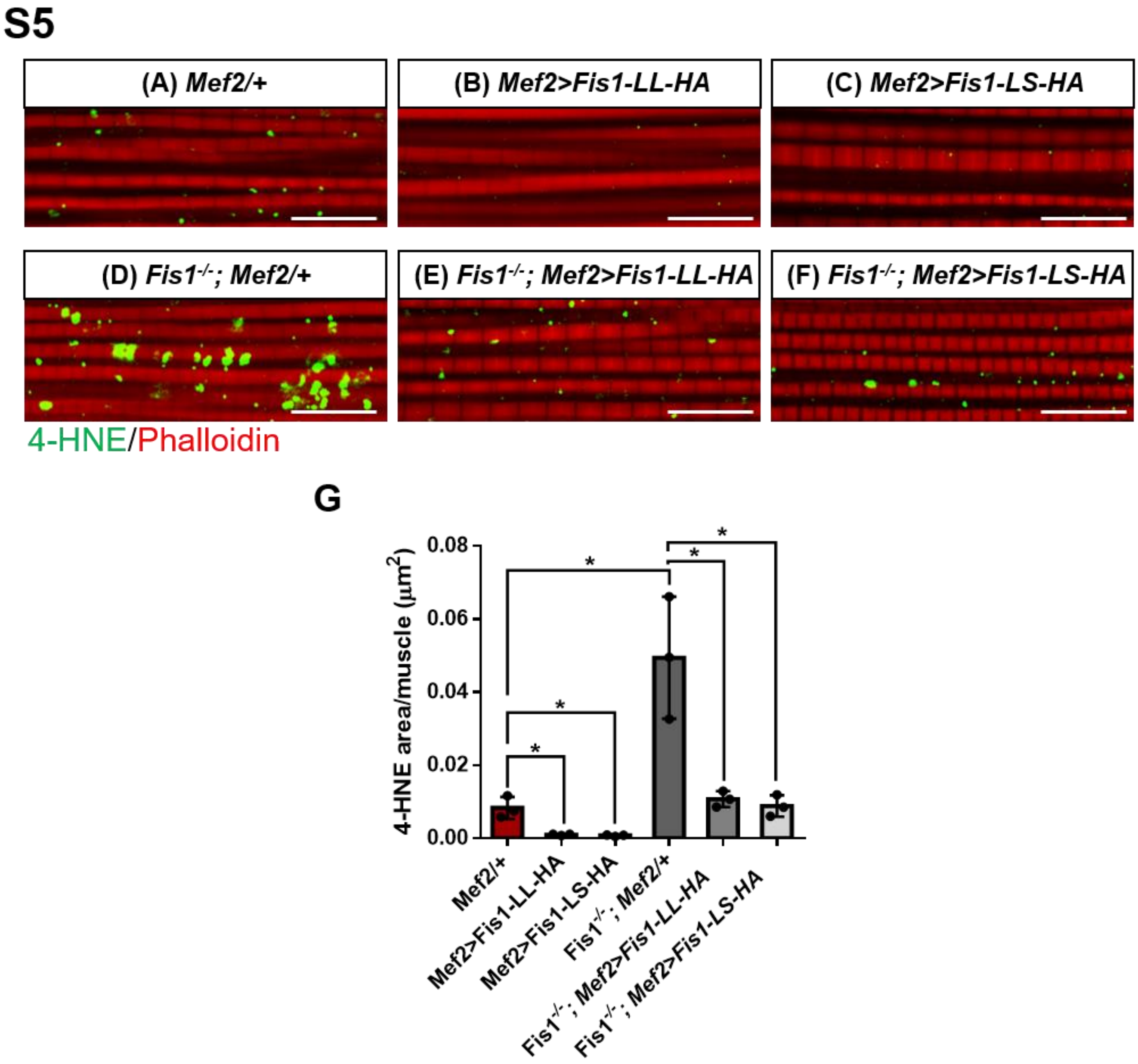
S5
(A) Mef2/+
(B) Mef2>Fis1-LL-HA
(C) Mef2>Fis1-LS-HA
(D) Fis1-/-; Mef2/+
(E) Fis1-/-; Mef2>Fis1-LL-HA
(F) Fis1-/-; Mef2>Fis1-LS-HA
4-HNE/Phalloidin
G
4-HNE area/muscle (µm2)
0.08
0.06
0.04
0.02
0.00
*
Mef2/+
Mef2>Fis1-LL-HA
Mef2>Fis1-LS-HA
Fis1-/-; Mef2/+
Fis1-/-; Mef2>Fis1-LL-HA
Fis1-/-; Mef2>Fis1-LS-HA

Figure S6

S6

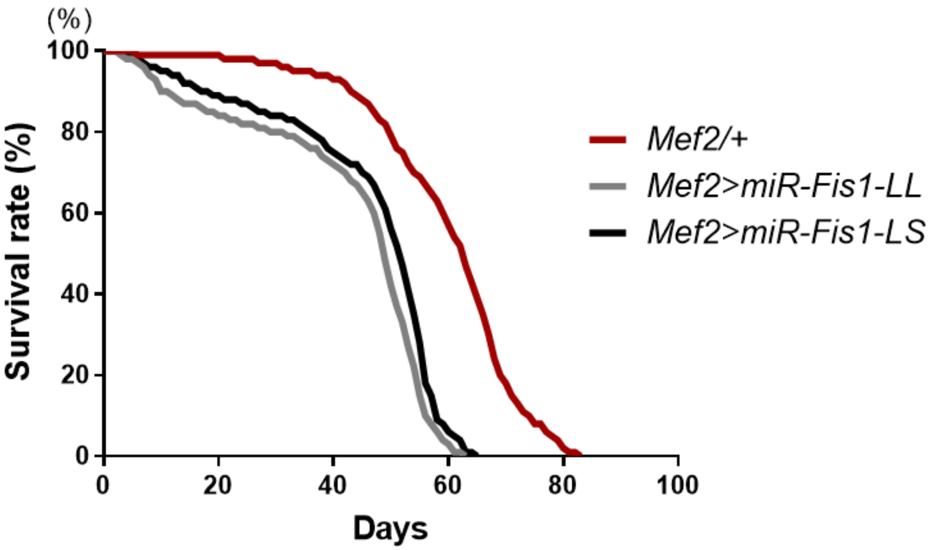
(%)
100
80
60
40
20
0
Survival rate (%)
0
20
40
60
80
100
Days
Mef2/+
Mef2>miR-Fis1-LL
Mef2>miR-Fis1-LS

Figure S7

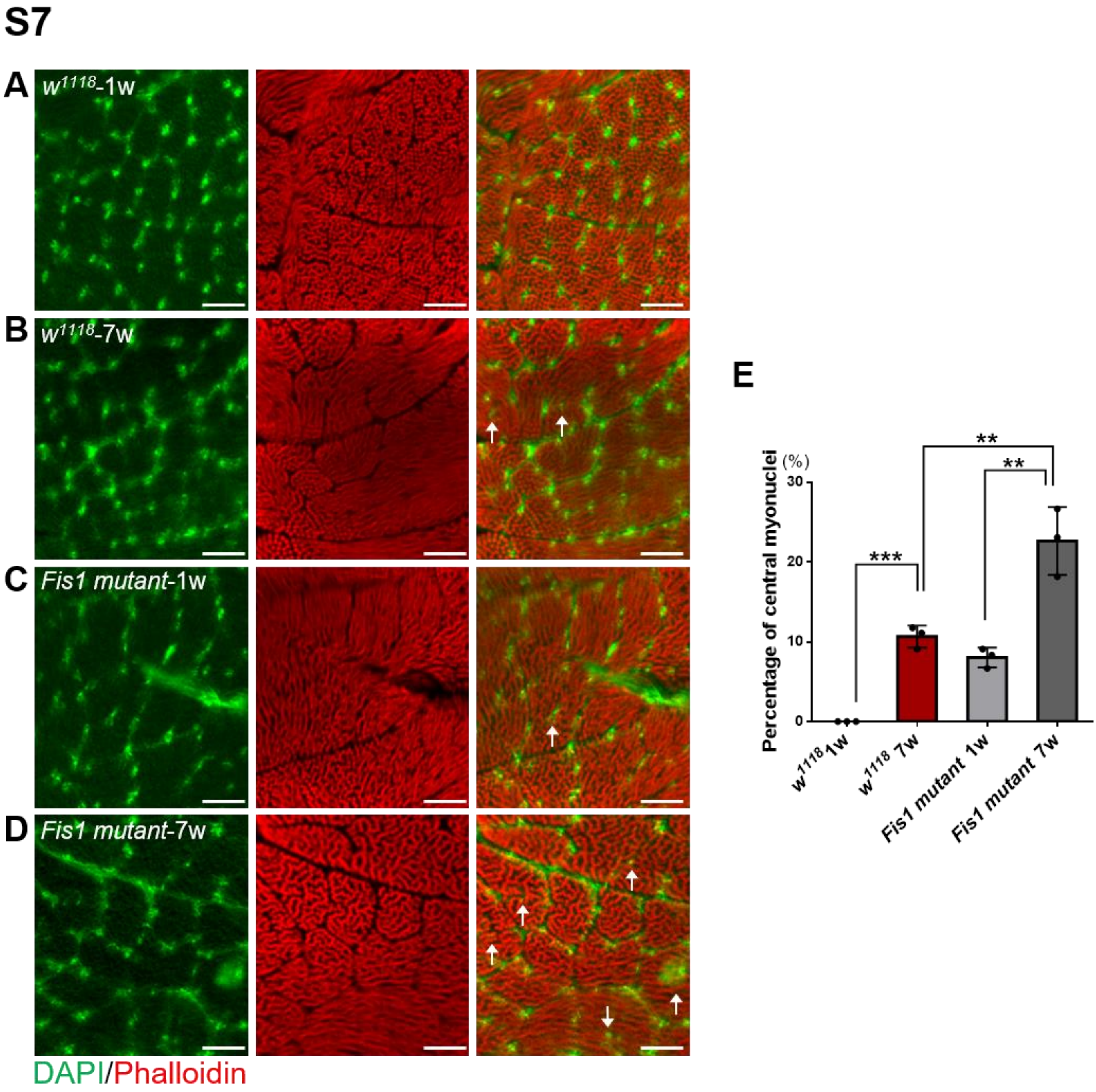

S7
A
w^{1118}-1w
B
w^{1118}-7w
C
Fis1 mutant-1w
D
Fis1 mutant-7w
DAPI/Phalloidin
E
Percentage of central myonuclei
(%)
30
20
10
0
***
**
**
w^{1118} 1w
w^{1118} 7w
Fis1 mutant 1w
Fis1 mutant 7w
